# Macro-Reticular Ion Exchange Resins for Recovery of Direct Dyes from Spent Dyeing and Soaping Liquors

**DOI:** 10.3390/molecules27051593

**Published:** 2022-02-28

**Authors:** Mona M. Naim, Nouf F. Al-harby, Mervette El Batouti, Mahmoud M. Elewa

**Affiliations:** 1 Chemical Engineering Department, Faculty of Engineering, Alexandria University, Alexandria 21526, Egypt; monanaim66@gmail.com; 2 Department of Chemistry, College of Science, Qassim University, Buraydah 51452, Saudi Arabia; 3 Chemistry Department, Faculty of Science, Alexandria University, Alexandria 21526, Egypt; 4 Arab Academy for Science, Technology and Maritime Transport, Alexandria P.O. Box 1029, Egypt; mahmoud.elewa@aast.edu

**Keywords:** direct dyes, macro-reticular ion exchange resins, adsorption isotherms, wastewater treatment, dye-house wastewater, recovery of dyes

## Abstract

Dyes are a major class of organic pollutants that are well-known for their harmful impact on aquatic life and humans. Several new strategies for removing colours from industrial and residential effluents have recently emerged, with adsorption being the best option. The current study looked at the recovery of direct dyes from aqueous streams for reuse using macro-reticular ion exchange resins (IERs). The investigation includes dyeing single jersey cotton grey textiles with direct dyes from the Isma dye Company in Kafr El Dawar, Egypt. After centrifuging and separating the supernatant liquid, solutions from thirteen different dyes, produced at an average concentration between the wasted and soaping liquor concentrations, were calculated spectrophotometrically from the first dyeing trials. Kinetic data were well fitted with pseudo-second-order rate kinetics. The amounts of dye retained by the anion exchangers increased with a rise in temperature in the case of Strong Base Resin (SBR) and vice versa for Weak Base Resin (WBR). Batch adsorption experiments with SBR and WBR were conducted for each dye, and both Freundlich and Langmuir isotherms were constructed. It was found that adsorption obeyed both isotherms, that monolayer adsorption took place, and that the dye molecular weight, structure, and solubility, as well as the type of anionic resin used, had varying effects on the extent of absorption. The monolayer sorption capacities *Q*_0_ determined from the Langmuir isotherm model for the strongly and weakly basic anion exchangers were found to be 537.6 and 692 mg/g for Direct Yellow RL, respectively. As a result, Yellow RL exhibited the greatest adsorption on both SBR and WBR. Orange GRLL, Blue 3B, and Congo Red, on the other hand, were the poorest colours absorbed by the IERs, whereas Blue RL demonstrated good adsorption by SBR and accelerated adsorption by WBR. Most of the dyes may be recovered and reused in this manner.

## 1. Introduction

The fast expansion in the global population and vast industrial operations has led global water demand to double every 21 years. Water issues affect almost 80 nations, accounting for 40% of the world’s population. According to the United Nations, water scarcity might affect up to 2.7 billion people by 2025 [1,2]. Furthermore, many countries have a scarcity of safe drinking water. Every year, around 5–10 million people die as a result of illnesses caused by drinking dirty water [1]. Water pollution control has been recently one of the major areas of scientific activity [3] The assessment of the amount of dye in the waste stream is an important investigation to do before disposal since even a tiny amount of 1 mg/L can cause colour and an unacceptable concentration for ingestion. Dyes are mostly organic complex molecules with the ability to connect to a variety of surfaces such as textiles, leathers, and so on. According to a recent analysis, there have been 1,000,000 commercially available dyes used all over the world, with a consumption record of 10,000 tons per year [4]. The fact that effluent is very complex in nature, along with this massive waste of textile dyes, causes the disturbance of an ecological system [5,6,7]. Dye-bearing effluents are a significant source of water pollution since they are used in numerous industries such as textile, printing, plastics, paper, carbide, food, and cosmetic industries [3,8]. Wastewaters (WWs) are generated predominantly in the following processes of textile production: slashing/sizing, de-sizing, scouring, bleaching, mercerizing, dyeing, printing, and finishing [9]. Their WWs also contain inorganic compounds such as chloride, carbonate, sulfate, phosphate, and heavy metals [5,10]. Since the discharge contains a variety of substances, each pollutant has a different toxicity threshold and poses a different hazard. However, nearly all of the compounds in the discharge stream are non-biodegradable in origin and have carcinogenic properties [11]. The presence of dye molecules in the hydrosphere inhibits sunlight penetration deep into water bodies, causing discomfort in the biological pursuit of aquatic life [12]. Toxicological effects have included growth inhibitions, reduced ingestion capacity, accumulation in living cells, increased enzymatic activities, decreased reproductive potential, kidney dysfunctions, protein level decrements, respiratory problems, opercular movements, histopathological variations, sub-lethal consequences, and so on. Not only have these impacts been highlighted, but many additional negative effects on aquatic and living species have been recorded as a result of toxic contaminants [13]. The environmental issues associated with residual colour in textile effluents had passed a major challenge to environmental scientists as well as to textile colouration processors. The requirement to remove the colour from textile effluents on-site prior to discharge to sewer has been progressively tightened due to increased public complaints about coloured water courses. Dyes are highly dispersible aesthetic pollutants and are difficult to treat, as most dyes are highly stable molecules made to resist degradation by light, chemical, biological, and other treatments or exposure [14]. They are mainly classified into cationic, anionic, and non-ionic dyes, from which the removal of anionic dye is considered the most challenging task since they are water-soluble and produce very bright colours with acidic properties [8]. A major contribution to colour in textile WW is the dyeing and the washing operation after dyeing, during which as much as 50% of the dye can be released into the effluent [14]. According to Mondal [15], dyes are constituents of textile WW that cause the largest difficulty in treatment. Ghaly et al. [9] give 10 to 250 mg/L as a range of dye concentrations in dye house effluents. Direct dyes are feasibly removed by biological treatment [16,17], adsorption [18,19,20,21], and ion exchange [22,23,24,25] as well as membrane processes, coagulation-flocculation [26,27], ozonation, and oxidation [28,29]. It has been estimated that the total dye consumption in the textile industry worldwide exceeds ten thousand tons per year, of which 10–15% of these dyes are released as effluents during the dyeing processes [28,30].

Effluent discharge from textile, leather and dye industries causes significant health concerns to environmental agencies [3] since their WW contains predominant dye substances that are toxic to the biological world; in addition, its dark colour locks sunlight, which leads to severe problems to the ecosystem through the eutrophication of aquatic eco-systems and serious health risk factors by bioaccumulation [28]. Accordingly, the decolourization of dyes is another important aspect of WW treatment prior to discharge into the environment.

Adsorption is the most often used technique for treating dye effluents [3,31]. Due to the range of adsorbents and the simplicity of operation, massive studies for dye removal have been published in the literature. There have been studies that have used activated carbon, activated carbon produced from biomass, carbon nanotubes, metal–organic frameworks, synthetic adsorbents, nanoparticles, and other materials [32]. The features of adsorbent, namely particular surface area, play an important role in the application. The greater the specific surface area, the greater the adsorption due to the porous spaces that absorb contaminants. Furthermore, various factors must be tuned during the process, including pH, dose, temperature, beginning concentration, and contact duration [33]. Due to charge attraction and Van der Waals forces, dye molecules have a high affinity for adsorption on the surface of adsorbents, resulting in chemical and physical adsorption [34]. According to studies, adsorbents have the best removal rates and sorption capacities while being easy to operate and economically practical [35]. The literature contains valuable information about the applicability of IERs in the removal of dyes from aqueous solutions [36,37,38,39,40,41,42,43]. Acid Orange 7 [44,45,46], Acid Orange 10 [45,47], Acid Green 9 [12], C.I. Acid Green 16 [48], Acid Red 18 [49], Acid Blue 29 [42], Acid Blue 113 [50], Basic Blue 3 [51], Tartrazine [13], Sunset Yellow [14], Reactive Black 5 [46], Reactive Orange 16 and Reactive Blue 21 [49], Reactive Red 120 [41] and 198 [17], Reactive Remazol Black B [52], Cationic Malachite Green [53], Direct Blue 71 [46,54], Direct Red 75 [55], Direct Yellow 50 [56,57], Congo Red [18], and other anion exchange resins have been shown to be very useful for the removal of acidic, basic, direct, and reactive dyes. Good sorption capabilities for the aforementioned dyes were discovered, as well as good regeneration.

In this work, a study on the removal and recovery of thirteen various direct dyes from dyeing and washing WWs was conducted by adsorption using macro-reticular strong-based IERs and weak-based IERs in batch experiments. Direct dyes outperform others in terms of cost, lightfastness, simplicity of application, and dye cycle length. So far, there is no comprehensive information in the literature on the use of anion exchange resins with various matrix compositions for direct dye removal. The kinetic and equilibrium parameters of the sorption process were determined, as well as the effect of time, concentration, pH, and temperature on dye adsorption efficiency. Then, both Freundlich and Langmuir isotherms were tested for their applicability, and the type of adsorption was determined.

## 2. Materials and Methods

### 2.1. Materials

#### 2.1.1. Direct Dyes

Thirteen direct dyes were obtained from Isma dye Company in Kafr El Dawar, Egypt, and were not purified prior to use. The trade name, structure, molecular formula, molecular weight, synonyms and wavelength of max absorbance of those dyes are presented in Table S1 in the Supplementary Materials section.

#### 2.1.2. Macro-Reticular Strong/Weak Base Anion Exchange Resins

AMBERLITE IRA958 Cl resin is a macro-reticular strongly basic anion exchange resin having quaternary ammonium functionality in a crosslinked acrylic polymer matrix. The porous macro-reticular structure allows the more efficient removal of large organic molecules and provides excellent resistance to physical breakdown by attrition and osmotic shock. The acrylic polymer structure contributes to the excellent desorption of organics during regeneration.

AMBERLITE IRA67 resin is a weak base anion exchange resin with a gel-type acrylic matrix. It has a high capacity, excellent physical stability, fast kinetics, outstanding resistance to organic fouling, and basicity higher than that of polystyrenic weak base resins.

The commercial synthetic, basic anion exchange resins with the acrylic skeleton: Amberlite IRA 67 of gel-type structure and Amberlite IRA 958-Cl of the macroporous structure were obtained from Rohm & Haas (France). The physicochemical properties and specifications of ion exchange resins are presented in Table 1.

**Figure 1:**
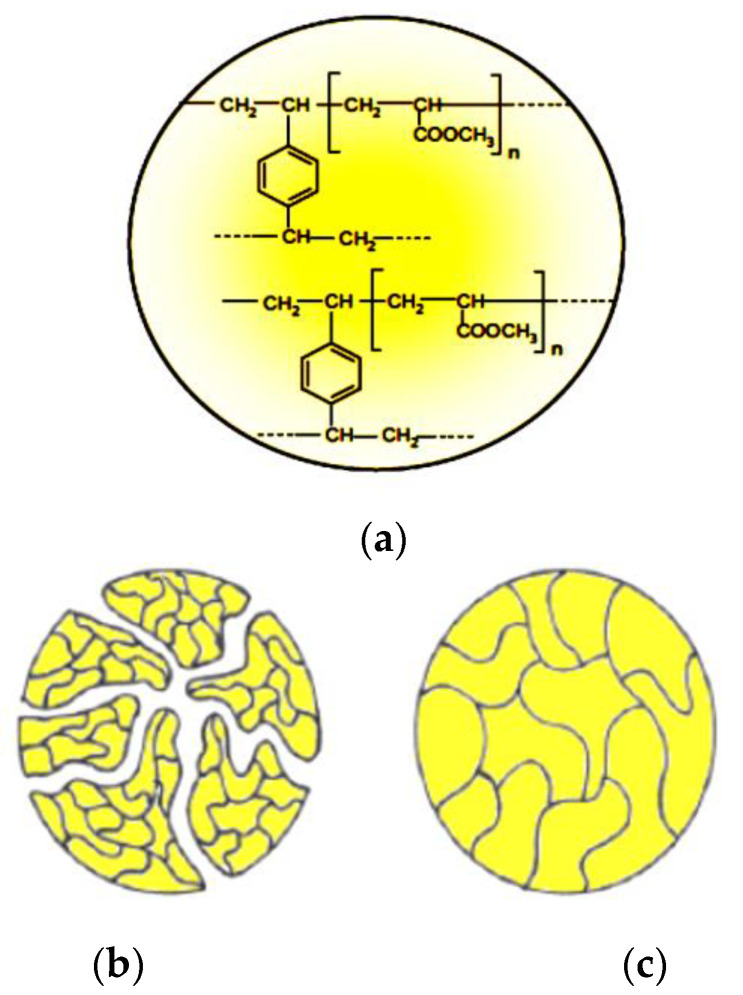
Composition of resin matrices: (**a**) acrylic-divinylbenzene skeleton, (**b**) macroporous, (**c**) gel [43].

Both Amberlite IRA 67and Amberlite IRA 958-Cl were chosen in the study on the dye removal because they are referred to as anion exchange resins and are particularly useful as an organic scavenger for effective adsorption of the naturally occurring organic molecules present in many water supplies. The matrix composition for both resins is acrylic-divinylbenzene (Figure 1a), and the structure for Amberlite IRA 958-Cl is macroporous (Figure 1b), while it is a gel (Figure 1c) for Amberlite IRA 67.

The resins were washed with distilled water to remove impurities and dried.

#### 2.1.3. Equipment and Chemicals

The solutions of all used dyes for adsorption experiments were analyzed using a model DR 5000™ UV-Vis spectrometer from Hach, Germany in 1 cm quartz cells using 200–1100 nm. The pH was measured with an HI 255 Combined Meter (pH/mV and EC/TDS/NaCl) from Hanna Instruments, Nijverheidslaan, Belgium. The mass of dyes and resin powder was weighed with Ohaus Adventurer Analytical Balance AX224M 220 g × 0.1 mg Internal Calibration, Scientific Laboratory Supplies, Nottingham, United Kingdom. The shaking of samples for solubility and adsorption were performed using Thermo Scientific™, MaxQ™ 4000 Benchtop Orbital Shakers Catalog number: SHKE4000-1CE. The dyeing of fabrics was performed in a Lab 2000mL (2L) Laboratory Electric Thermostatic & Adjustable Heating Mantle, HEQI GLASS.

### 2.2. Methods

#### 2.2.1. Dyeing Experiments

**Figure 2:**
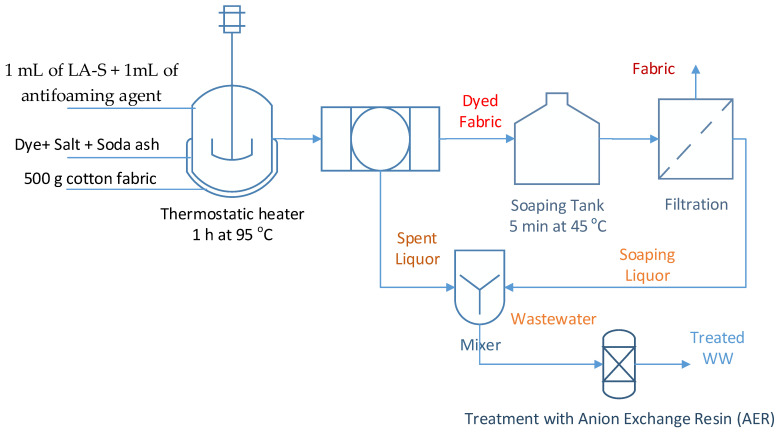
Schematic of dyeing and soaping fabrics for preparing dyed WW.

Single jersey cotton grey fabrics (500 g) were dyed with direct dyes from Isma dye Company, Kafr El Dawar Egypt, at a liquor to a good ratio of 1:10, at 95 °C in a thermostatic heater, for 60 min, in the presence of 1 mL of LA-S and 1 mL of the antifoaming agent. The dye bath recipe was according to the Isma dye colour shade card (Figure 2). Table 2 describes the dyeing experimental conditions.

The spent liquors were separated, and the dyed fabrics were soaped with 500 mL water at 45 °C for 5 min. The spent and soaping liquors were separated and compared with other solutions of known concentration using a spectrophotometer, and their average concentrations were computed.

#### 2.2.2. Determination of Calibration Curve

Various dyes of known concentrations were prepared, and their absorbance values were measured by a spectrophotometer. Then, the calibration curves of each dye were drawn, from which the exact concentrations could be estimated via the slope of the absorbance–concentration lines obtained.

#### 2.2.3. Centrifugation Experiments

From the previous dyeing experiments, solutions from the various dyes were prepared at an average concentration between the spent and soaping liquors concentrations estimated previously. The solutions were centrifuged for one hour at room temperature at 3750 rpm and a relative centrifugal force of 3600 gravities. The supernatant liquid was separated, and the dye concentration was determined. The saturation concentration of the various dyes was calculated at room temperature.

#### 2.2.4. Kinetic Experiments

First, 100 mL of each dye solution at a respective initial dye concentration varying from 100 to 500 mg/L was added to 0.5 g of the resin in a small tightly closed glass-stoppered bottle. Five of these bottles were shaken by a thermostatic shaker at 180 rpm [58] for different time intervals (0–180 min). The contents of each bottle were decanted with care in a dry clean beaker. A sample was analysed to determine the concentration of the dye spectrophotometrically at a specific wavelength for each dye at which absorption is maximum. Plots of concentration versus time were constructed, and the minimum time required to reach equilibrium was determined for each dye. The amount of dye adsorbed a time *t* (*q_t_* (mg/g)) was calculated from Equation (1) [43,54,59]:

(1) $$q_{t}=\frac{\left(C_{0}-C_{t}\right)}{m}V$$

The amount of adsorption at equilibrium, $q_{e}$ (mg/g), was calculated by:

(2) $$q_{e}=\frac{\left(C_{0}-C_{e}\right)}{m}V$$

where $C_{0}$ is the dye output concentration mg/L, $\;C_{t}$ is the concentration after sorption time *t* (mg/L), $C_{e}$ is the dye concentration in the equilibrium state mg/L, V is the solution volume L, and *m* is the resin mass g.

For the description of experimental data, two popular models were applied, based on which the kinetic parameters were calculated: pseudo-first-order model (PFO) and pseudo-second-order model (PSO).

**Pseudo-First-Order Model (PFO):** The Langergren [60] model from 1898 of the first order is determined by the following Equation (3):

(3) $$Log\left(q_{e}-q_{t}\right)=Logq_{e}-\frac{k_{1}}{2.303}t$$

where $k_{1}$ is the pseudo-first-order rate constant (1/min), *t* is time (min), $q_{e}$, $q_{t}$ are the amounts of dye adsorbed at equilibrium and after time *t* (mg/g), respectively.

From the slopes and intercepts of the plots log $\left(q_{e}-q_{t}\right)$ vs. *t*, $k_{1}$ and $q_{e}$ were calculated.

**Pseudo-Second-Order Model (PSO):** This model proposed by Ho and McKay [61,62] in 1998 is represented by the following Equation (4):

(4) $$\frac{t}{q_{t}}=\frac{1}{k_{2}q_{e}^{2}}+\frac{1}{q_{e}}t$$

where $k_{2}$ is the pseudo-second order rate constant (g/mg·min), *t* is time (min), $q_{e}$, $q_{t}$ are the amounts of dye adsorbed at equilibrium and after time *t* (mg/g), respectively.

If the kinetics of the sorption process are described by the PSO model, the *t*/$q_{t}$ vs. *t* graph is linear, and $q_{e}$ d $k_{2}$. can be determined from the slope and intercept.

#### 2.2.5. Equilibrium Experiments

Adsorption isotherms are used to determine the balance between the concentration of adsorbate in the solid phase and its concentration in the liquid phase. Based on the course of the isotherm, information about the maximum adsorption capacity of the sorbent can be obtained. The most commonly used models are Langmuir and Freundlich ones. Different weights of resins were separately added to a glass-stoppered bottle followed by adding 100 mL of one dye liquor. The bottle was shaken for the predetermined optimum time for each dye, which was sufficient to reach equilibrium. The temperature was recorded and controlled in each case. Then, the contents of the bottles were decanted in a clean, dry beaker, and a sample of each dye was analysed for its concentration at its optimum wavelength. The experiments were conducted using both SBRs and WBRs.

**Langmuir Model:** The Langmuir isotherm equation describes chemical adsorption. The adsorbed substance forms a monomolecular film on the surface of the solid phase. The Langmuir adsorption isotherm is the basic and most widespread adsorption equation that can be considered as the initial equation for a number of more detailed studies [63,64,65]. The linear form of the Langmuir isotherm is given below (5):

(5) $$\frac{C_{e}}{q_{e}}=\frac{1}{Q_{0}b.}+\frac{C_{e}}{Q_{0}}$$

where $C_{e}$ is the equilibrium dye concentration (mg/L), $Q_{0}$ is the monolayer capacity (mg/g), *b* is the Langmuir constant (L/mg), and $q_{e}$ is the amount of dye adsorbed at equilibrium (mg/g).

The values of the Langmuir parameters were obtained from the plots $C_{e}$/$q_{e}\;\mathrm{vs}.\;1/C_{e}$.

A characteristic feature of the Langmuir isotherm is the dimensionless separation factor $R_{L}$, which can be calculated as follows (6):

(6) $$R_{L}=\frac{1}{1+bC_{0}}$$

where $C_{0}$ is the highest initial dye concentration (mg/L), and *b* is the Langmuir constant (L/mg).

In addition, the value of the dimensionless $R_{L}$ coefficient determines the shape of the isotherm: unfavorable ($R_{L}$ > 1), linear $R_{L}$( = 1), favorable (0 < $R_{L}$ < 1), or irreversible ($R_{L}$ = 0) [18,63].

**Freundlich model:** The Freundlich (1906) isotherm equation describes the well adsorption on heterogeneous surfaces (non-uniform energy) and microporous adsorbents [59]. It is used for description of reversible adsorption and is not confined to the formation of a single layer. The Freundlich model can be described based on the following Equation (7):

(7) $$\log q_{e}=\log K_{F}+\frac{1}{n}\log C_{e}$$

where $K_{F}$ is the Freundlich constant, $\frac{1}{n}$ is the parameter characterizing the energy heterogeneity of the adsorbent surface, $C_{e}$ is the equilibrium dye concentration (mg/L), and $q_{e}$. is the amount of dye adsorbed at equilibrium (mg/g). The values of the Freundlich parameters were calculated from the plots $\log q_{e}$. vs. $\log C_{e}$. The value $\frac{1}{n}$ > 1 indicates a weak bond between the adsorbate and adsorbent molecules, while a value of $\frac{1}{n}$ < 1 points to a strong adsorption bond as a result of strong intermolecular attractions in the adsorbent layers [64,66].

#### 2.2.6. Effect of Solution pH

By mixing the matching anion exchanger (0.5 g) with the dye solution (C_0_ = 1 g/L) at the requisite pH, the effect of the initial pH of the dye solution changing from 1 to 12 on Direct Yellow RL sorption utilizing WBR and SBR was investigated. Dilute 0.1 M NaOH and 0.1 M HCl solutions were used to alter the pH. Decantation was used to remove the solution from the anion exchanger, and the dye concentration was evaluated by analysing the absorbance value spectrophotometrically.

#### 2.2.7. Effect of Temperature 

Mixing the matching anion exchanger with the dye solution (C_0_ = 250–18,000 mg/g) for 24 h at three different temperatures, 25, 35, and 45 °C, was used to investigate the effect of temperature on Direct Yellow RL sorption utilizing SBR and WBR. The samples were taken out, the solution was filtered out of the anion exchanger, and the concentration was evaluated using an absorbance value analysis.

## 3. Results and Discussion

### 3.1. Determination of Calibration Curve

Using a UV-Vis spectrometer from Hach Lange, Germany, the maximum absorbance of Direct Yellow RL is 398 nm is shown in Figure 3. The data for calibration curves and maximum absorbances for all direct dyes studied in the present work are presented in Table 3. The calibration curve of Yellow RL dye is depicted in Figure 4. All the calibration curves for the remaining 12 direct dyes in this study are depicted in the Supplementary Materials section (Figures S1–S12).

### 3.2. Determination of Solubilities

Solubilities of various direct dyes in water were determined according to the methodology mentioned in Section 2.2.3. Table 4 shows the solubilities of the various dyes used in the present investigation.

### 3.3. Effect of Phase Contact Time

**Figure 5:**
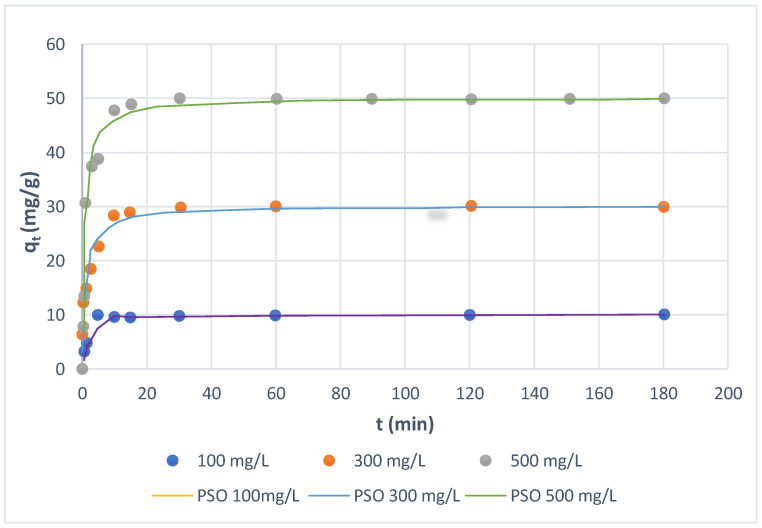
Effects of phase contact time and initial concentration of Direct Yellow RL on the amount of dye adsorbed by SBR.

**Figure 6:**
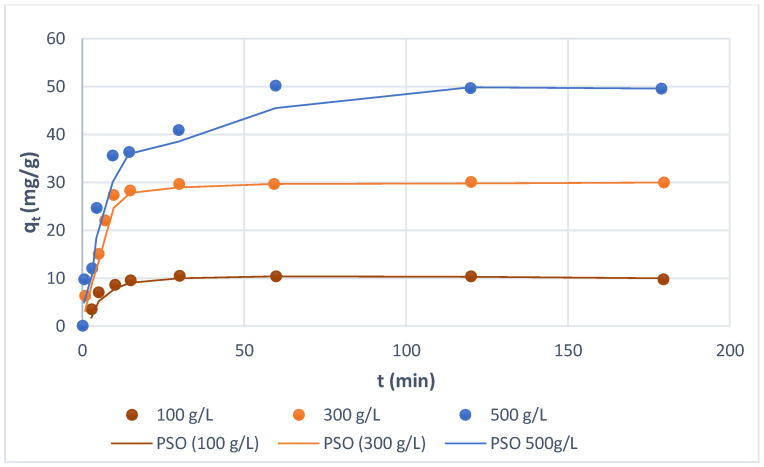
Effects of phase contact time and initial concentration of Direct Yellow RL on the amount of dye adsorbed by WBR.

The kinetic dependencies of the studied 13 direct dyes adsorption on Amberlite SBR and WBR were measured for various initial dye concentrations (100–500 mg/L) at room temperature. As examples, the dependencies of the amounts of the dye adsorbed on the anion exchanger, *q_t_*, vs. the phase contact time are shown in Figure 5 and Figure 6 for Direct Yellow RL dye. As can be observed, at the specified concentrations, the quantity of dye adsorbed grew fast at first, then linearly at a slower rate, and finally, there was no growth. The reached saturation, which is referred to as the equilibrium time, depends on the concentration of dye. For the macroreticular polyacrylic anion exchanger of the quaternary functionalities Amberlite IRA 958, the phase contact time needed to reach equilibrium was 4.7 min in the aqueous solution containing 100 mg/L of dye, while for higher initial concentrations of 300 and 500 mg/L of dye, it was 15.8 and 30 min, respectively (Figure 5). For the weakly basic tertiary amine gel anion exchanger Amberlite IRA 67, the time required for the complete uptake of Yellow RL dye was slower, and for initial concentrations of 100, 300, and 500 mg/L, it was 14.8, 29.8, and 60 min, respectively, as shown in Figure 6. Wawrzkiewicz [55] reported a similar result in the sorption of C.I. Direct Red 75 from an aqueous solution on weakly basic (Amberlite IRA67) and strongly basic (Amberlite IRA458) anion exchangers of the polyacrylic matrix. The time it took to reach equilibrium increased as the dye concentration in the water phase increased; it took 30 min, 60 min, and 120 min for Amberlite IRA67 and 40 min, 60 min, and 120 min for Amberlite IRA458 at initial concentrations of 100 mg/L, 500 mg/L, and 1000 mg/L, respectively.

### 3.4. Sorption Kinetic Parameters

Knowledge of adsorption kinetics allows for the adjustment of the entire adsorption process to improve its efficacy. The process of Direct Yellow RL dye adsorption on SBR and WBR was described using two commonly used models: pseudo first order (PFO) and pseudo second order (PSO). These equations are based on the amount of material adsorbed by a given mass unit of adsorbent in a given time unit. Based on the projected kinetic parameters, which are reported in Table 5, it is possible to determine which model is the most compatible.

#### 3.4.1. Pseudo-First-Order (PFO) Model

**Figure 7:**
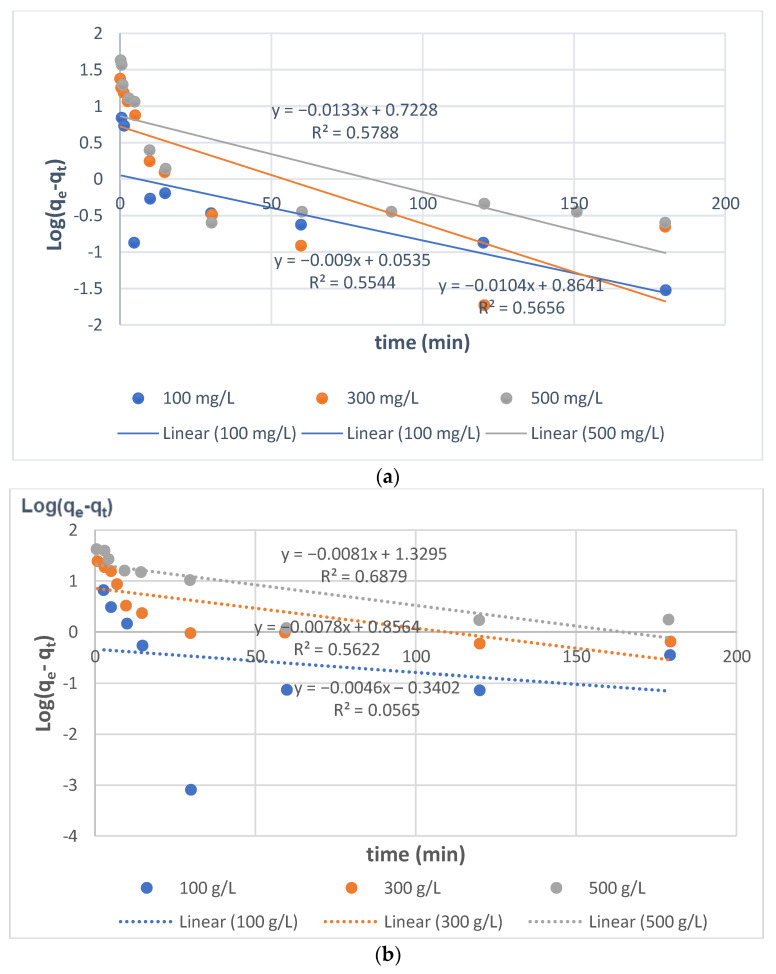
Log $\left(q_{e}-q_{t}\right)$. vs. *t* dependencies determined from the linear form of the PFO model in (**a**) Yellow GL-SBR and (**b**) Yellow GL-WBR systems.

Due to the small values of the determination coefficients R^2^ in the ranges 0.554–0.579 for SBR and 0.056–0.688 for WBR, the Lagergren equation was not used to explain the sorption kinetics of Direct Yellow RL and all direct dyes in the current study on anion exchange resins of diverse matrices such as Amberlite IR 958 and Amberlite IR 67. Furthermore, the estimated equilibrium capacities were much lower than those obtained experimentally (Figure 7 and Table 5). Furthermore, the log $\left(q_{e}-q_{t}\right)$ vs. *t* graph was not linear.

#### 3.4.2. Pseudo-Second-Order (PSO) Model

**Figure 8:**
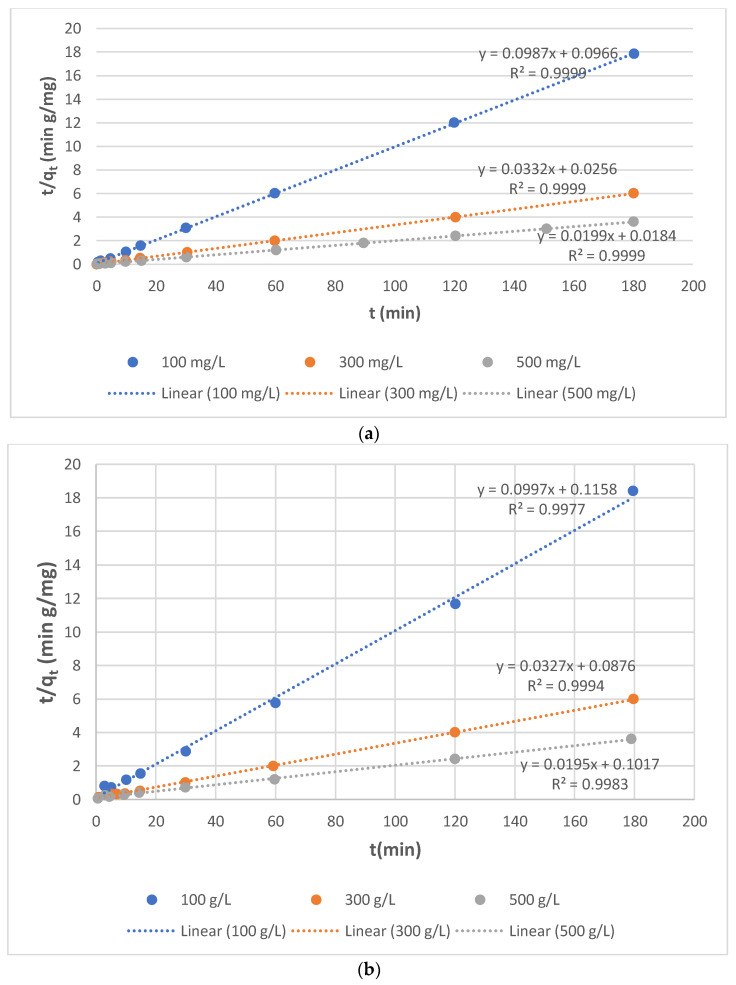
*t*/$q_{t}$. vs. *t* dependencies determined from the linear form of the PSO model in (**a**) Yellow GL-SBR and (**b**) Yellow GL-WBR systems.

Figure 8 depicts the linearized form of the PSO kinetic dependence for Yellow RL sorption from different initial concentrations of solution on SBR and WBR. If the kinetics of the sorption process are described using the PSO model, the plot $t/q_{t}$. vs. *t* yields a linear relationship from which $q_{e}$. and k_2_ can be calculated. The k_2_ constant drops as the concentration of dye in the aqueous solution grows in the pseudo-second-order model. Based on the data in Table 5 and Figure 7 and Figure 8, it is conceivable to conclude that the PSO model better matched the experimental data. This is demonstrated not just by high R^2^ determination coefficient values (0.999).

### 3.5. Adsorption Isotherms

**Figure 9:**
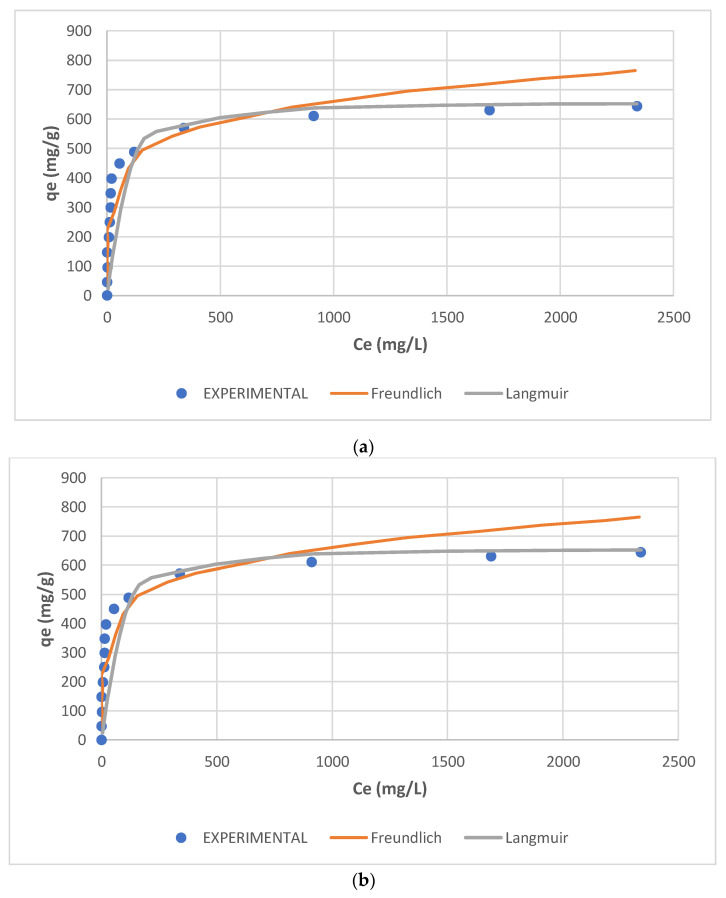
Equilibrium uptake of Direct Yellow RL retention by (**a**) SBR, (**b**) WBR, and the fitting of Freundlich and Langmuir isotherm models.

According to Brunauer [67], the types of isotherms obtained in the present work are in most cases those of type I, which are those associated with systems where adsorption does not proceed beyond the monomolecular layer, whereas other shapes are of the types that involve multi-layer formation. Plots of X/m ($q_{e}$) versus C_e_ plotted for all the dyes, only Yellow GL shown here (Figure 9), indicate that many of the dyes investigated are of the mono-layer type e.g., Yellow RL and Red 8B. Similar observations can be concluded for Turquoise Blue GLL, Blue 3B, Blue 4GL, Brown RC and Blue RL, while others are of the multi-layer type II, as e.g., Yellow G, Orange GRLL, and Congo Red, which in addition are isotherms of the unfavourable type. However, some other dyes such as Black Meta are highly adsorbed from dilute solution by SBR, whereas they are poorly adsorbed by WBR. As to Orange GRLL, its isotherms are unfavourable with both resins, whereas adsorption is almost doubled in the case of SBR, while in the case of Yellow G, adsorption is weak with both types of resins and Blue 3B is adsorbed only from dilute solutions in both cases due to its restricted solubility at 23 °C. The figures show that monolayer adsorption follows both isotherms. On the other hand, it is observed in both cases that Blue 4GL is poorly adsorbed and that IE is probably not a good option for its recovery. The figures also indicate that the dye’s bulk structure, configuration, and poor solubility are controlling factors in determining the poor extent of adsorption onto both SBR and WBR.

From the aforementioned results, it can be deduced that Congo Red and Violet R are the worst dyes adsorbed by the AERs among the dyes tested, whereas Yellow RL and Red 8B exhibit favourable adsorption by SBR and moderate adsorption by WBR (at only dilute solutions in case of the last dye).

#### 3.5.1. Freundlich Isotherms

**Figure 10:**
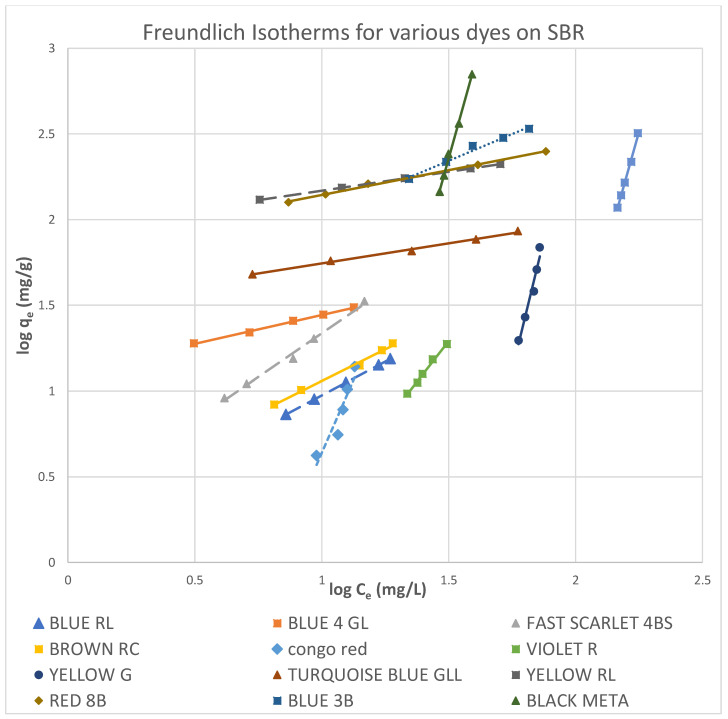
Freundlich isotherms for various dyes on SBR.

**Figure 11:**
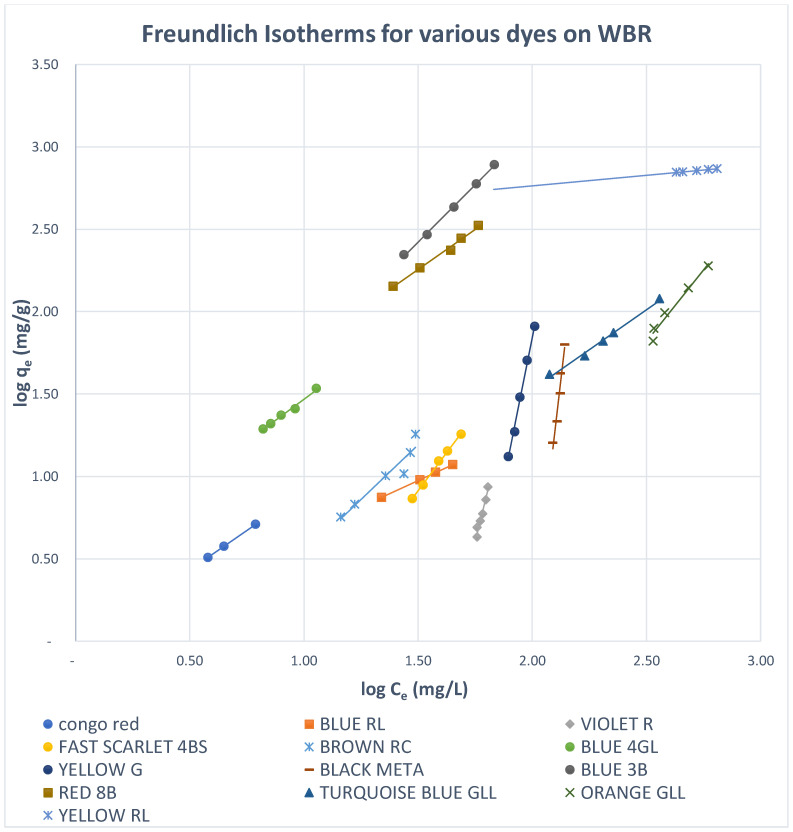
Freundlich isotherms for various dyes on WBR.

**Figure 12:**
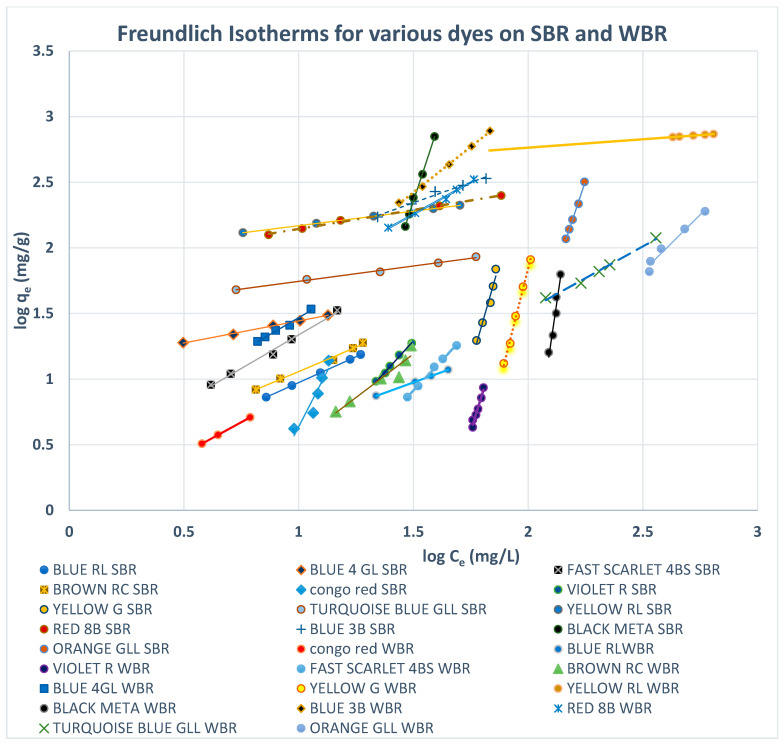
Freundlich isotherms for various dyes on SBR and WBR.

**Figure 13:**
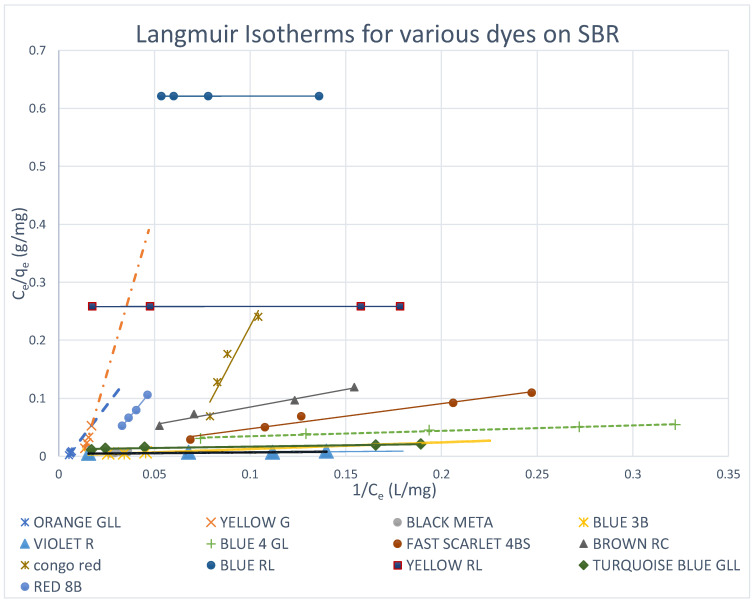
Langmuir isotherms for various dyes on SBR.

**Figure 14:**
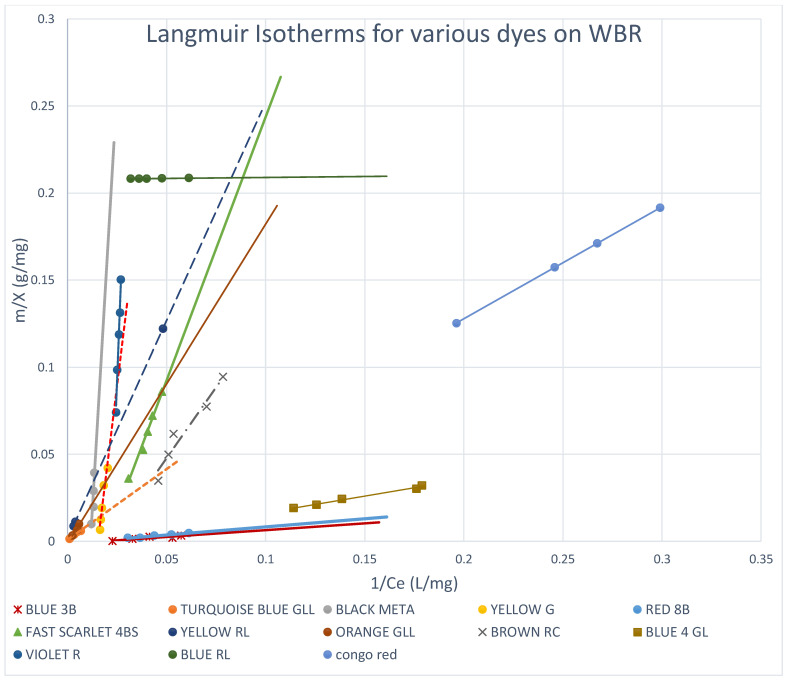
Langmuir isotherms for various dyes on WBR.

**Figure 15:**
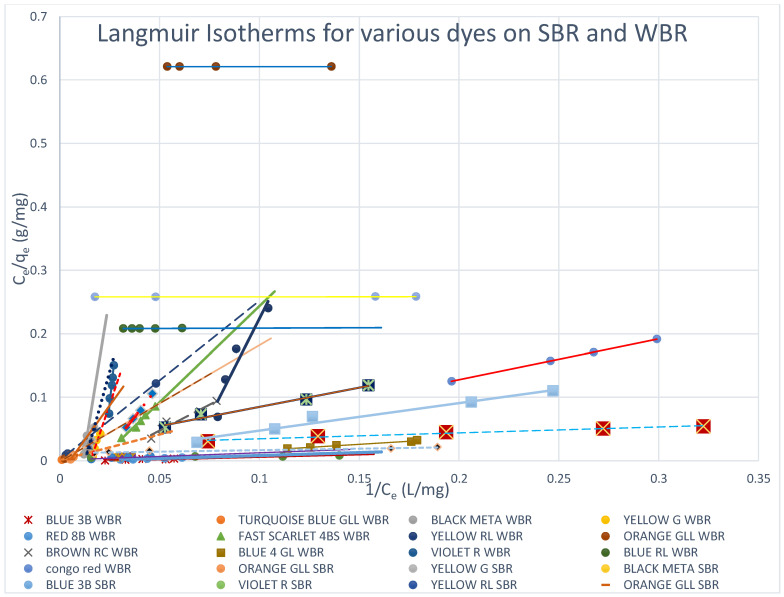
Langmuir isotherms for various dyes on SBR and WBR.

Both Freundlich and Langmuir isotherms were plotted as shown in Figure 10, Figure 11, Figure 12, Figure 13, Figure 14 and Figure 15, in which the experiments were conducted mostly at 23 °C. Figure 10 and Figure 11 present the Freundlich isotherms for all dyes tested, using SBR and WBR in respective order. From Figure 10, Yellow RL and Red 8B are the best dyes removed by SBR, while Yellow G is the weakest. Again, from Figure 11, it is noticed that the isotherms obtained using WBR are all steeper than those in Figure 10. However, Yellow RL, Red 8b, Blue 4GL, and Blue RL are the best adsorbed, but still, they are the least adsorbed by WBR similar to the case of SBR. Table 6 presents the values of Log *K_F_* (intercept) and 1/n (slope) in the Freundlich equation for the different dyes treated in the present work. The higher the value of *K_F_*, the better the dye removal of the resin. On the other hand, the lower the value of 1/n, i.e., the more an isotherm tends to be horizontal, the better the dye removal at all dye concentrations tested. According to Equation (7), the slope (1/n) is parameter characterizing the energy heterogeneity of the adsorbent surface. The direct dyes where the value $\frac{1}{n}$ > 1 indicates a weak bond between the adsorbate and adsorbent molecules from Table 6, in the case of SBR are Yellow G, Black Meta, Orange GRLL, Congo Red, Violet R, and Scarlet 4BS, while a value of $\frac{1}{n}\;$< 1 points to a strong adsorption bond as a result of strong intermolecular attractions in the adsorbent layers [64,66], which in the case of SBR includes Yellow RL, Turquoise GLL, Red 8B, Blue 4GL, Blue 3B, Brown RC, and Blue RL.

Moreover, from Table 6, it is observed that the dyes that adsorb best by SBR with values of $\frac{1}{n}\;$< 1 are yellow RL, Blue RL, Congo red, Turquoise GLL, and Red 8B. The dyes where the values of $\frac{1}{n}\;$> 1 are Black Meta, Yellow G, Violet R, Scarlet 4BS, Orange GRLL, Blue 3B, Brown RC, and Blue 4GL. As regards WBR, the table indicates that *K_F_* values are unacceptable in most cases except Yellow RL, Blue RL, and Red 8B.

The values of K_F_ in the case of Yellow RL adsorbed by SBR and WBR were 88.79 and 321.66 (mg^1−1/n^ L^1/n^/g), respectively. Similar results were obtained by Wawrzkiewicz et al. [56] in the adsorption of C.I. Direct Yellow 50 by strong base Amberlite IRA 958 Cl resin. The values of *K_F_* for Blue RL were 0.6501 and 1.005 (mg^1−1/n^ L^1/n^/g), respectively, and almost the same results were attained by [54] C.I. Direct Blue 71 removal from aqueous solutions and wastewaters by strong base Amberlite IRA 958 Cl and weak base Amberlite IRA 67.

Figure 12 shows this remark clearly since it presents the Freundlich isotherms for both SBR and WBR on the same plot for comparison. Overall, it could be stated that adsorption onto SBR is much more favourable than WBR, proving that the macro-reticular SBR resin can effectively adsorb the direct dyes tested in the present work compared to WBR.

#### 3.5.2. Langmuir Isotherms

The Langmuir adsorption model implies that all adsorption sites are comparable, that no interactions exist between adsorbed molecules and neighbouring sites, and that adsorption occurs in a monolayer. In contrast to the Langmuir model, the Freundlich model is applicable to heterogeneous sorption on surfaces with several types of sites. The Langmuir isotherms have been plotted for all the dyes on both SBR and WBR, as illustrated in Figure 13 and Figure 14, while the isotherms for the various dyes on SBR and WBR are presented together on one plot for comparison in Figure 15. The computed constants for the Langmuir model were heavily influenced by the kind of anion exchanger (Table 7). The basicity of the resin, as well as the matrix composition and structure, were discovered to be deciding variables of sorption. The best monolayer sorption capacities calculated using the Langmuir equation for Amberlite IRA 958 and Amberlite IRA 67 were found to be 537.63 mg/g (R^2^ = 0.999) and 692 mg/g (R^2^ = 0.828), respectively, for Yellow RL and 1610.32 mg/g (R^2^ = 0.9999) and 89.1 mg/g (R^2^ = 0.9999), respectively, for Blue RL. The dimensionless constant separation factor *R_L_* [36] was used to investigate the effect of isotherm shape on whether adsorption is favourable or unfavourable. It was discovered that *R_L_* values in the 0–1 range indicate the Yellow RL dye’s preferential uptake by SBR (*R_L_* = 0.480769) and WBR (*R_L_* = 0.4878048). It is observed that the Langmuir isotherms are obeyed in all the cases studied (*R_L_* ≈ 0–1) as indicated in Table 7, which proves that adsorption is only mono-layer, as expected. This could be explained as follows: the dye is attracted to adsorption sites after diffusing through the resin pores, where it reacts with the resin’s positively charged sites internally as well as on the surface, and it prevents further layers from being attached due to the repulsion of free sulfonic and carboxylic acid groups on the adsorbed dye molecule, which prevents further adsorption onto the initial monolayer.

### 3.6. Impact of Quantity of Amberlite IRA Resins

**Figure 16:**
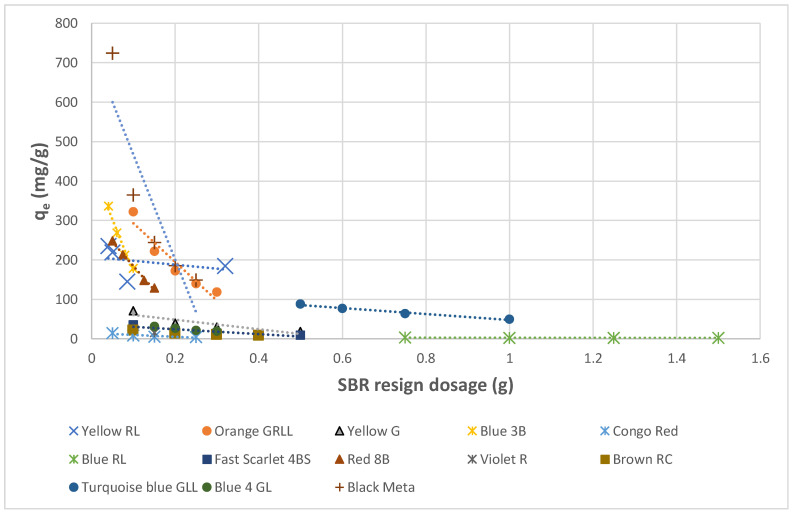
Impact of Amberlite IRA 958 Cl resin quantity on the adsorption of direct dyes.

**Figure 17:**
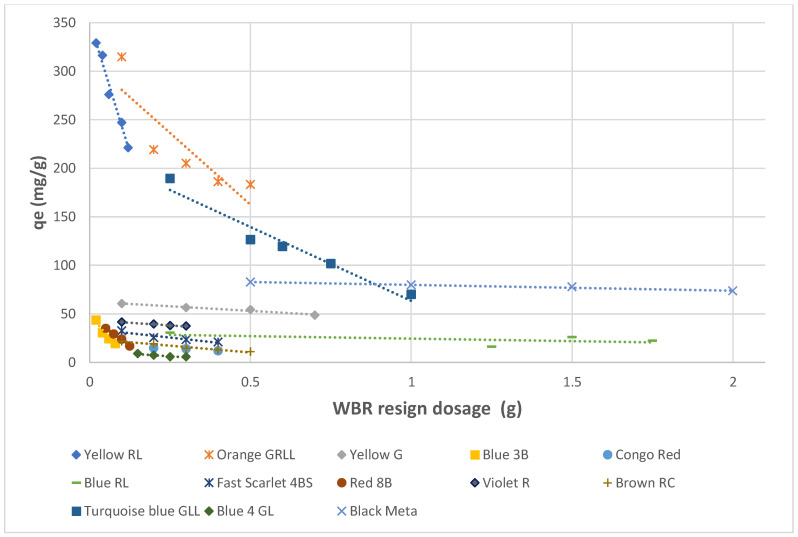
Impact of Amberlite IRA 67 resin quantity on the adsorption of direct dyes.

The impact of the quantities of Amberlite IRA resin on the acrylic acid adsorption was investigated by replacing different amounts of 0.02–2 g for each Amberlite IRA resin by using the concentration of dyes that resulted from dyeing and soaping illustrated in Table 8 at the equilibrium contact time of 240–600 min and a temperature of 20–21 °C. The results of these adsorption experiments were illustrated in Figure 16 and Figure 17. They show an abrupt decrease in the capacity of adsorption with the increasing amount of the adsorbents. It has been observed from Figure 16 and Figure 17 that as the resin quantity rose, the adsorption capacity values for both Amberlite IRA decreased prominently for some direct dyes and slightly for others. However, the lowest dosage for both Amberlite resins can be considered as the optimum adsorbent dosage in this interval studied. By using this optimum adsorbent quantity, the highest adsorption capacity values were 724.52 mg/g for Amberlite IRA-958 in case of Black Meta and 329 mg/g for Amberlite IRA-67 in case of Yellow RL.

### 3.7. Effects of Initial pH

The acidity or basicity of the solution has a big impact on the ion exchange process. The ionic form of the resin (dissociation degree of functional groups), and consequently the nature of interactions between a dye and an adsorbent, is affected by the pH of the solution. In the pH range of 2 to 10, the direct dye chosen (Yellow RL) for the investigations has four sulfonic groups in ionized form. WBR has tertiary amine groups that work at low pH when the hydrogen ion concentration is high enough to protonate the resin, whereas SBR has quaternary ammonium groups that are ionic in both acid and basic environments, and both sorption capacities stay constant throughout a wide pH range. The dye adsorption on both resins was unaffected by the solution pH. After 15 min of phase contact time, the *q_t_* values for SBR and WBR were 47.9 and 45.8 mg/g, respectively. Non-electrostatic mechanisms, as well as traditional ion exchange mechanisms, should be considered to explain this behaviour [58,68]. There can be hydrogen bonding interactions between the nitrogen of the tertiary amine groups and the oxygen of the dye’s hydroxyl group. The van der Waals force being hydrophobic interaction, i.e., the π-π interaction between the aromatic character of the polymeric matrix and the aromatic ring of the dye, can also be ascribed to WBR’s acceptable performance [54,55,58,68]. The former is a more relevant adsorption process for hydrophilic and highly water-soluble direct dye molecules that exist as anions in the aqueous phase throughout a wide pH range.

The pH value of the solution, which is a key regulating parameter in adsorption, is determined primarily by two factors: (i) the distribution of the dye ionized species in the solution phase, and (ii) the total charge of the adsorbent. As a result, the interaction between the dye molecules and the adsorbent is essentially a combination of charges on the dye molecules and the adsorbent’s surface [69,70].

### 3.8. Effects of Initial Temperature

**Figure 18:**
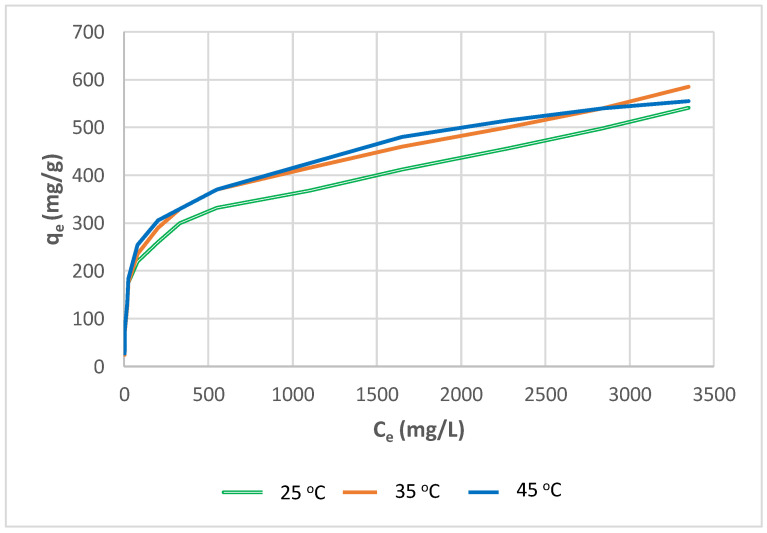
Experimental adsorption isotherms of Direct Yellow RL for SBR achieved at various temperatures (conditions: phase contact time: 180 min, volume: 20 mL, resin mass: 200 mg, agitation rate: 180 rpm).

**Figure 19:**
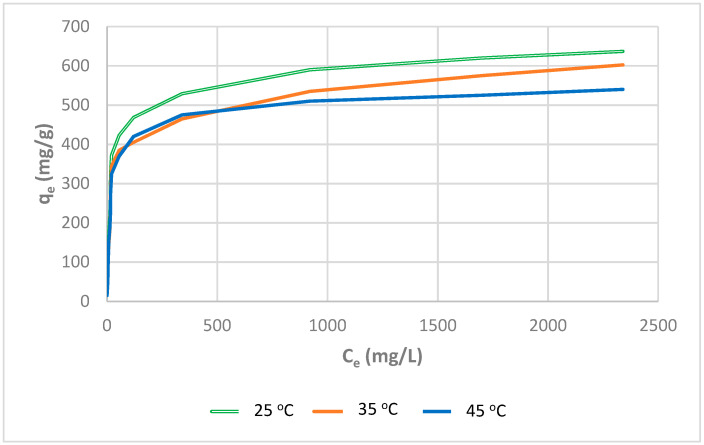
Experimental adsorption isotherms of Direct Yellow RL for WBR achieved at various temperatures (conditions: phase contact time: 180 min, volume: 20 mL, resin mass: 200 mg, agitation rate: 180 rpm).

Since certain textile dye effluents are generated at high temperatures, temperature can be an essential issue in the practical application of anion exchangers [71]. The influence of temperature on the equilibrium Yellow RL dye adsorption capacity of each anion exchanger was studied in the temperature range of 25–45 °C at various initial dye concentrations, with a constant quantity of adsorbent of 0.2 g and a constant contact period of 180 min. Figure 18 and Figure 19 show graphs of the dye quantity adsorbed at equilibrium (*q_e_*) vs. the liquid-phase concentrations of the dye at equilibrium (C_e_) at three examined temperatures for the Yellow RL-SBR and Yellow RL-WBR systems, respectively. In the instance of dye adsorption on Amberlite IRA-958, there is a significant increase in Direct Yellow RL absorption when the temperature is elevated from 25 to 45 °C. Temperature increases have a detrimental influence on the adsorption process, and dye absorption decreases insignificantly. The variation in total adsorption capacity of SBR for Direct Yellow RL between 25 and 45 °C is about 45 mg/g. The increase in adsorption might be explained by a reduction in the thickness of the boundary layer surrounding the resin beads as the temperature rises, lowering the dye molecules’ mass transfer resistance in the boundary layer. This might be due to an increase in the dye molecules’ mobility as their kinetic energy rises as well as a faster rate of intraparticle diffusion of the adsorbate as the temperature rises [70]. A closer look at Yellow RL adsorption behaviour on Amberlite IRA-958 at a higher temperature of 45 °C reveals that the adsorption capacity value of 560 mg/g obtained at this temperature is lower than the extremely high values of 592 mg/g achieved at 35 °C. Greluk and Hubicki observed a similar temperature influence on the dye adsorption trend for Reactive Black 5 adsorption on the strongly basic anion exchanger Amberlite IRA-958 [68]. This behaviour might be explained by a reversible adsorption process or a reverse diffusion control mechanism.

The adsorption capability of Amberlite IRA-67 decreased significantly with increasing temperature, from 640 to 535 mg/g for a temperature range of 25–45 °C. This suggests that the exothermic mechanism regulated the adsorption of Yellow RL on the weakly basic WBR at a low temperature of 25 °C. The slopes of the isotherms at 35 and 45 °C are shallow, indicating that interactions between the dye and the weakly basic Amberlite IRA-67 were minimal at these temperatures. The observed results can be attributed to the previously described poor attraction of tertiary amine groups to dye anions. As a result, as the temperature rises, the dye anions may re-emerge from the solid phase into the bulk phase. Furthermore, when the temperature rises, the solubility of the direct dye rises, and the contact forces between the molecule and the solution become higher than those between the molecule and the anion exchanger, making the dye more difficult to adsorb [72,73]. The decrease in the anion exchanger adsorption capacity for direct dyes with increasing temperature is consistent with the findings of Tan et al. [63] for Methylene Blue adsorption on coconut husk-based activated carbon and Vimonses et al. [74] findings for a study evaluating the adsorption capacity of Australian clay materials to remove Congo Red.

When the obtained adsorption capacity values for both anion exchangers, weakly basic Amberlite IRA-67 and strongly basic Amberlite IRA-458, are compared, it is clear that Amberlite IRA-958 has significantly better adsorption properties for direct dyes adsorption over the entire temperature range investigated. It follows that the quaternary ammonium groups of SBR have a stronger affinity for the dye anions than the tertiary amine groups of low basicity resins, such as WBR.

## 4. Conclusions

It has been shown from the present work that direct dyes can be recovered from aqueous solution to different extents, by both macroreticular SBR and WBR, depending on the structure, configuration, molecular size, number and type of solubilizing groups, and the type of resin: whether SBR or WBR. The predominating factors seem to be the dye molecular weight and the type of resin (SBR or WBR), followed by the type and number of solubilizing groups, then lastly the dye configuration. The equilibrium data were fitted well using the Langmuir isotherm model. The conclusions may be stated as follows:

- The dye molecular weight is a predominating factor in controlling its diffusion through the resin pores to the active adsorption sites, and therefore, it greatly influences the degree of adsorption that takes place.
- The number of solubilizing groups in the dye molecule, and their type (whether -SO_3_H or -COOH), affects the degree of adsorption and controls the extent of adsorption to different anion exchangers.
- The type of resin, whether strong base or weak base anion exchanger, affects the extent of dye adsorption.
- Macro-reticular resin has proven to be efficient in the adsorption of different dyes compared to the other type.
- The number and type of substituent groups in the dye molecule affect the degree of adsorption to the resin.

## Figures and Tables

**Figure 3:**
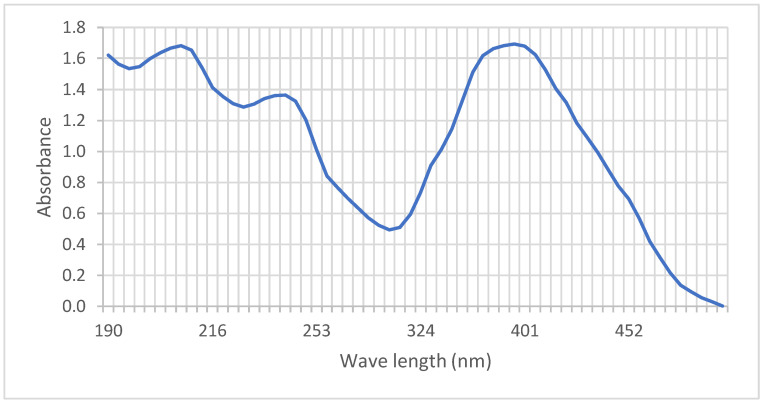
Absorbance vs. wavelength for Direct Yellow RL; the maximum absorbance is 396 nm.

**Figure 4:**
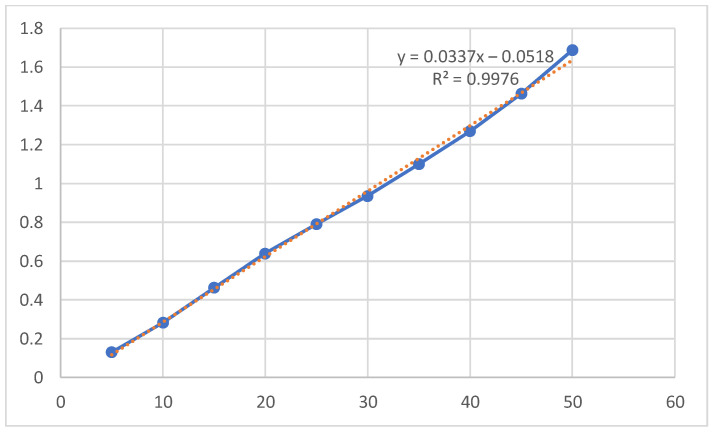
Calibration curve of Yellow RL dye (5–50) mg/L, pH 7, 25 °C.

**Table 1:** Properties of Amberlite IRA 958-Cl [43,56] and Amberlite IRA67 [43,58].

Anion Exchanger Properties	Amberlite IRA 958-Cl	Amberlite IRA 67
Physical form	White spherical beads	White, translucent, spherical beads
Matrix	Crosslinked acrylic macroreticular structure	Crosslinked acrylic gel structure
Functional group	Quaternary ammonium	Tertiary amine—N(CH_3_)_2_
Ionic form	Chloride—N^+^ (CH_3_)_3_ Cl^−^	Free Base (FB)
Matrix composition and structure	acrylic-divinylbenzene, macroporous	acrylic-divinylbenzene, gel
Moisture-holding capacity	66 to 72% (Cl-form)	56 to 64% (FB form)
Particle size:		
Uniformity coefficient Harmonic mean size	≤1.8 630–850 µm	≤1.80 500–750 µm
Molecular formula		C_30_H_44_N_2_O_4_
Molecular weight		496.68100
BET surface area	2.03 m^2^/g	
Total exchange capacity	≥0.8 eq/dm^3^	>1.6
Temperature limitations	80 °C	60 °C
Operating pH range	0–14	0–7
Density (g/cm^3^)	0.2097	0.2367

**Table 2:** Dyeing experimental conditions.

Dye Trade Name	Conc. (g/L)	NaCl (g)	Soda Ash (g)
Yellow RL	1	5	-
Orange GRLL	1.25	10	-
Yellow G	1.25	10	-
Blue 3B	1.25	10	-
Congo red	1	10	1
Blue RL	1.5	10	-
Scarlet 4BS	1	10	0.5
Red 8B	1.25	5	-
Violet R	0.5	5	-
Brown RC	1	20	-
Turquoise GLL	2	10	-
Blue 4GL	1	5	-
Black Meta	4	10	-

**Table 3:** Calibration curves data.

Dye Trade Name	Slope	Intercept	R^2^	Wavelength Used (λ_max_)
Yellow RL	0.0337	−0.0518	0.9976	398
Orange GRLL	0.0139	0.0601	0.999	444
Yellow G	0.0245	0.0068	0.9999	497
Blue 3B	0.0258	0.099	0.9823	608
Congo Red	0.039	−0.0635	0.9968	502
Blue RL	0.0149	0.1737	0.9992	588
Scarlet 4BS	0.0267	0.0123	0.9994	507
Red 8B	0.0454	0.0296	0.9997	510
Violet R	0.0277	0.0386	0.9993	532
Brown RC	0.0559	−0.0166	0.9992	460
Turquoise GLL	0.0005	0.0007	0.9984	600
Blue 4GL	0.0427	0.021	0.9997	605
Black Meta	0.0465	0.0216	0.9987	520

**Table 4:** Solubilities of various dyes.

Dyestuff Trade Name	Solubility at Room Temperature (g/L)
Yellow RL	0.4071
Congo Red	0.0269
Blue 3B	0.294
Yellow G	0.1676
Blue RL	0.0451
Scarlet 4BS	0.0522
Violet R	0.0666
Brown RC	0.0475
Turquoise Blue GLL	0.7286
Blue 4GL	0.0579
Black Meta	0.8788
Orange GRLL	0.5198
Red 8B	0.0556

**Table 5:** Kinetic parameters for adsorption of Direct Yellow RL on IER using the pseudo first order and pseudo second order.

Resin	C_0_ (mg/L)	Experimental *q_e_* (mg/g)	PFO			PSO		
			*q_e_* (mg/g)	K_1_ (1/min)	R^2^	*q_e_* (mg/g)	K_2_ (g.mg/min)	R^2^
Amberlite IRA 958 Cl (SBR)	100	9.99	0.979	0.123	0.554	10.13	0.1008	0.9999
	300	28.6	0.976	1.990	0.566	30.12	0.0431	0.9999
	500	50	0.736	1.664	0.579	50.25	0.0215	0.9999
Amberlite IRA 67 (WBR)	100	9.49	0.457	0.0106	0.056	10.03	0.0858	0.9997
	300	29.64	7.184	0.0179	0.562	30.58	0.0122	0.9994
	500	50.09	21.35	0.0186	0.688	51.28	0.0037	0.9983

**Table 6:** Equations, values of slope, and intercept for Freundlich isotherms.

**Direct Dye Trade Name**	**Strong Base Resin (Amberlite IRA 958CL)**				
	**Equation of the Straight Line**	**(Intercept) (Log *K_F_*)**	**(Slope)** **(1/n)**	**R^2^**	$K_{F}$ **(mg** **^1^** **^−^** **^1/n^ L** **^1/n^** **/g)**
Yellow RL	y = 0.2204x + 1.9484	1.9484	0.2204	0.9985	88.79
Orange GRLL	y = 5.2708x − 9.348	−9.348	5.27085	0.9958	4.48 × 10^−10^
Yellow G	y = 6.0946x − 9.5475	−9.5475	6.0946	0.9621	2.834 × 10^−10^
Blue 3B	y = 0.624x + 1.4071	1.4071	0.624	0.9819	25.53
Congo Red	y = 3.3993x − 2.7613	−2.7613	3.3993	0.8785	0.00173
Blue RL	y = 0.7873x + 0.1870	0.1870	0.7873	0.9997	0.6501
Scarlet 4BS	y = 1.0183x + 0.318	0.318	1.0183	0.9919	2. 079
Red 8B	y = 0.2896x + 1.8542	1.8542	0.2896	0.9965	71.48
Violet R	y = 1.8972x − 1.5557	−1.5557	1.8972	0.9949	0.0278
Brown RC	y = 0.7382x + 0.32	0.32	0.7382	0.9922	2.0892
Turquoise GLL	y = 0.2343x + 1.5101	1.5101	0.2343	0.9948	32.366
Blue 4GL	y = 0.3362x + 1.1069	1.1069	0.3362	0.9974	12.79
Black Meta	y = 5.3139x − 5.615	−5.615	5.3139	0.9968	2.43× 10^−6^
**Direct Dye Trade Name**	**Weak Base Resin (Amberlite RA 67)**				
	**Equation of the Straight Line**	**K** **(Intercept) (Log *K_F_*)**	**n** **(Slope) (1/n)**	**R^2^**	$K_{F}$ **(mg** **^1^** **^−^** **^1/n^ L** **^1/n^** **/g)**
Yellow RL	y = 0.1280x + 2.5074	2.5074	0.128	0.9989	321.66
Orange GRLL	y = 1.7365x − 2.5227	−2.5227	1.7365	0.9687	0.003
Yellow G	y = 6.9428x − 12.043	−12.043	6.9428	0.9963	9.57 × 10^−13^
Blue 3B	y = 1.3913x + 0.335	0.3919	1.3913	0.9987	2.465
Congo Red	y = 0.9578x − 0.046	−0.046	0.9578	1	0.899
Blue RL	y = 0.6357x +0.0216	0.00216	0.6357	0.9999	1.005
Scarlet 4BS	y = 1.85x − 1.8614	−1.8614	1.85	0.9977	0.0137
Red 8B	y = 0.9715x + 0.7985	0.7985	0.9715	0.9921	6.287
Violet R	y = 5.677x − 9.3312	−9.3312	5.677	0.9641	4.66 × 10^−10^
Brown RC	y = 1.3269x − 0.7979	−0.7979	1.3269	0.914	0.1592
Turquoise GLL	y = 0.9676x − 0.4082	−0.4082	0.9676	0.9926	0.3907
Blue 4GL	y = 1.0246x + 0.4431	0.4431	1.0246	0.9876	2.7739
Black Meta	y = 11.932x − 23.772	−23.772	11.932	0.9426	1.69 × 10^−24^

**Table 7:** Equations, values of slope, and intercept for Langmuir isotherms.

**Direct Dye Trade Name**	**Strong Base Resin (Amberlite IRA 958CL)**						
	**Equation of the Straight Line**	**(Intercept)** **(1/*Q*_0_ b)**	**(Slope) (1/*Q*_0_)**	**R^2^**	***Q*_0_ (mg/g)**	**b (L/mg)**	***R_L_*** **from Equation (6)**
Yellow RL	y = 0.00186x + 0.258	0.2583	0.00186	0.9999	537.634	0.0072	0.480769
Orange GRLL	y = 4.3446x − 0.0212	−0.0212	4.3446	0.9888	0.23017	−204.93	−9.759 × 10^−6^
Yellow G	y = 11.299x − 0.1417	−0.1417	11.299	0.9886	0.08850	−79.738	−8.361 × 10^−5^
Blue 3B	y = 0.1149x + 0.0011	0.0011	0.1149	0.9275	8.70322	104.45	4.786 × 10^−5^
Congo Red	y = 6.2127x − 0.3971	−0.3971	6.2127	0.9094	0.16096	−15.645	−0.003206
Blue RL	y = 0.00062x + 0.62099	0.62099	0.00062	0.9999	1610.32	0.0001	0.9900990
Scarlet 4BS	y = 0.4338x + 0.0042	0.0042	0.4338	0.9678	2.30520	103.28	0.0001936
Red 8B	y = 4.0172x − 0.0812	−0.0812	4.0172	0.9959	0.24892	−49.472	−0.0001010
Violet R	y = 0.0259x + 0.0043	0.0043	0.0259	0.8749	38.6100	6.0232	0.0033095
Brown RC	y = 0.6064x + 0.0244	0.0244	0.6064	0.9793	1.64907	24.852	0.0010049
Turquoise GLL	y = 0.0473x + 0.012	0.012	0.0473	0.9081	21.1416	3.9416	0.0005071
Blue 4GL	y = 0.0932x + 0.0254	0.0254	0.0932	0.9747	10.7296	3.6692	0.0045217
Black Meta	Y = 0.2934x − 0.0032	−0.0032	0.2934	0.9942	3.40831	−91.687	−2.7267 × 10^−5^
**Direct Dye Trade Name**	**Weak Base Resin (Amberlite RA 67)**						
	**Equation of the Straight Line**	**(Intercept) (1/*Q*_0_ b)**	**(Slope) (1/*Q*_0_)**	**R^2^**	***Q*_0_**	**b (L/mg)**	***R_L_*** **from Equation (6)**
Yellow RL	y = 2.50650x + 0.00144	2.50650	0.00144	0.9932	692	0.007	0.4878048
Orange GRLL	y = 1.8352x − 0.0014	−0.0014	1.8352	0.9423	0.544899	−1310.	−1.526 × 10^−6^
Yellow G	y = 9.2704x − 0.1425	−0.1425	9.2704	0.9481	0.107870	−65.055	−0.0001024
Blue 3B	y = 0.0781x − 0.0014	−0.0014	0.0781	0.7777	12.80409	−55.785	−8.963 × 10^−5^
Congo Red	y = 0.6434x − 0.0009	−0.0009	0.6434	0.9998	1.554243	−714.88	−6.994 × 10^−5^
Blue RL	y = 0.0112x + 0.2078	0.20784	0.0112	0.9999	89.1	0.054	0.15625
Scarlet 4BS	y = 3.0257x − 0.0589	−0.0589	3.0257	0.9866	0.330502	−51.370	−0.0003894
Red 8B	y = 0.0933x − 0.001	−0.001	0.0933	0.9524	10.71811	−93.3	−5.359 × 10^−5^
Violet R	y = 29.184x − 0.6376	−0.6376	29.184	0.9797	0.034265	−45.771	−0.0004371
Brown RC	y = 1.6284x − 0.0337	−0.0337	1.6284	0.9459	0.614099	−48.320	−0.0005176
Turquoise GLL	y = 0.8126x + 0.0009	0.0009	0.8126	0.9486	1.230617	902.88	2.215 × 10^−6^
Blue 4GL	y = 0.1918x − 0.0028	−0.0028	0.1918	0.9896	5.213764	−68.5	−0.0002433
Black Meta	y = 19.301x − 0.2238	−0.2238	19.301	0.8572	0.051810	−86.242	−2.89 × 10^−5^

**Table 8:** Experimental conditions for batch experiments.

Dye Trade Name	Dye Conc. (g/L)	Resin Type	Resin Weight (g)	Shaking Time (min)	Temperature (°C)
Yellow RL	0.15	SBR	0.04, 0.05, 0.07, 0.1	240	21
Orange GRLL	0.5	SBR	0.1, 0.15, 0.2, 0.25, 0.3	240	21
Yellow G	0.15	SBR	0.1, 0.2, 0.3, 0.5	240	21
Blue 3 B	0.2	SBR	0.04, 0.06, 0.08, 0.1	240	21
Congo Red	0.02	SBR	0.05, 0.1, 0.15, 0.25	240	21
Blue RL	0.04	SBR	0.75, 1.0, 1.25, 1.5	240	21
Scarlet 4BS	0.05	SBR	0.1, 0.2, 0.3, 0.4, 0.5	240	21
Red8B	0.2	SBR	0.05, 0.075, 0.125, 0.15	240	21
Violet R	0.05	SBR	0.1, 0.15, 0.2, 0.3	240	21
Brown RC	0.04	SBR	0.1, 0.2, 0.3, 0.4	240	21
Turquoise Blue GLL	0.5	SBR	0.5, 0.6, 0.75, 1.0	240	21
Blue 4GL	0.06	SBR	0.15, 0.2, 0.25, 0.3	240	21
Black Meta	0.4	SBR	0.05, 0.1, 0.15, 0.2, 0.25	240	21
Yellow RI	0.15	WBR	0.02, 0.04, 0.06, 0.1, 0.12	600	20
Orange GRLL	0.5	WBR	0.1, 0.2, 0.3, 0.4, 0.5	600	20
Yellow G	0.15	WBR	0.1, 0.3, 0.5, 0.7	600	20
Blue 3B	0.2	WBR	0.02, 0.04, 0.06, 0.08	600	20
Congo Red	0.025	WBR	0.2, 0.3, 0.4	600	20
Blue RL	0.04	WBR	0.25, 0.5, 0.75, 1.25	600	20
Scarlet 4 BS	0.05	WBR	0.1, 0.2, 0.3, 0.4	600	20
Red 8B	0.2	WBR	0.05, 0.075, 0.1, 0.125	600	20
Violet R	0.05	WBR	0.1, 0.2, 0.25, 0.3	600	20
Brown RC	0.04	WBR	0.1, 0.2, 0.3, 0.4, 0.5	600	20
Turquoise Blue GLL	0.05	WBR	0.25, 0.5, 0.6, 0.75, 1.0	600	20
Blue 4GL	0.06	WBR	0.15, 0.2, 0.25, 0.3	600	20
Black Meta	0.4	WBR	0.5, 1.0, 1.5, 2.0	600	20

## Data Availability

Not applicable.

## Supplementary Materials

The following are available online. Figure S1: Calibration curve of Orange GRLL dye (30–300) mg/L, pH 7, 25 °C, Figure S2: Calibration curve of Blue 3B dye (10–80) mg/L, pH 7, 25 °C, Figure S3: Calibration curve of Congo Red dye (10–100) mg/L, pH 7, 25 °C, Figure S4: Calibration curve of Red 8B dye (1–50) mg/L, pH 7, 25 °C, Figure S5: Calibration curve of Scarlet 4BS (5–75) mg/L, pH 7, 25 °C, Figure S6: Calibration curve of Violet R (10–100) mg/L, pH 7, 25 °C, Figure S7: Calibration curve of Brown RC (1–50) mg/L, pH 7, 25 °C, Figure S8: Calibration curve of Turquoise Blue GLL (10–800) mg/L, pH 7, 25 °C, Figure S9: Calibration curve of Blue RL (5–45) mg/L, pH 7, 25 °C, Figure S10: Calibration curve of Yellow G (1–100) mg/L, pH 7, 25 °C, Figure S11: Calibration curve of Blue 4GL (5–60) mg/L, pH 7, 25 °C, Figure S12: Calibration curve of Black meta (5–20) mg/L, pH 7, 25 °C, Table S1: Trade Name, Structure, Molecular Formula, Molecular Weight, Synonyms, and Wavelength of max absorbance of direct dyes, Table S2: Freundlich isotherm for SBR data, Table S3: Freundlich isotherm for WBR data, Table S4: Langmiur isotherm for SBR data, Table S5: Langmiur isotherm for WBR data.
