# Supplementary material for: Macro-Reticular Ion Exchange Resins for Recovery of Direct Dyes from Spent Dyeing and Soaping Liquors
Source: Molecules. 2022 Feb 28;27(5):1593. doi: 10.3390/molecules27051593 (PMC8912055; doi:10.3390/molecules27051593)
Supplement: Supplementary file 1 [file molecules-27-01593-s001.zip › molecules-1578540-supplementary.pdf]

Supplementary Information

Table S1: Trade Name, Structure, Molecular Formula, Molecular Weight, Synonyms, and Wavelength of max absorbance of direct dyes

Dye Trade Name	Dye Structure	Molecular Formula	Molecular Weight (g/mol)	Synonyms	Wavelength of max absorbance (nm), λ_{max}	CAS NO.
Yellow RL	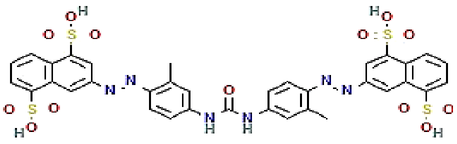	$C_{35}H_{28}N_6O_{13}S_4$	868.9	C.I. Direct Yellow 50, tetrasodium salt	396	3214-47-9
Orange GRLL	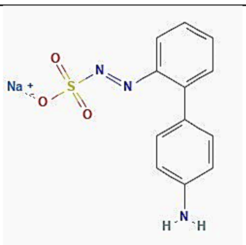	$C_{12}H_{10}N_3NaO_3S$	299.28	Direct Orange 39 CI 40215	444	1325-54-8
Yellow G	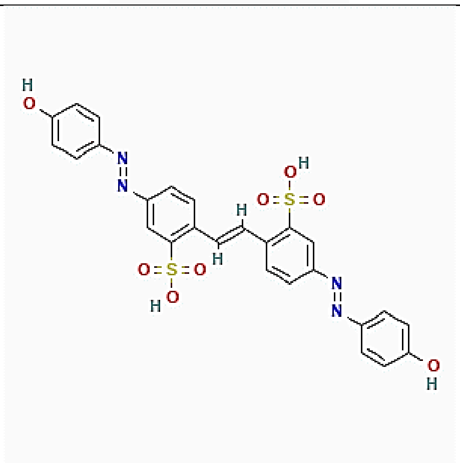	$C_{26}H_{20}N_4O_8S_2$	580.6	C.I. Direct Yellow 4, disodium salt	497	3051-11-4
Blue 3B	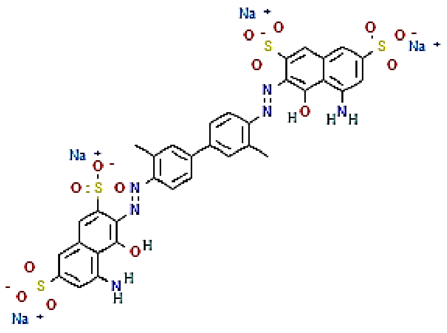	$C_{34}H_{24}N_6Na_4O_{14}S_4$	960.8	Trypan Blue C.I. 23850	608	72-57-1

Congo red	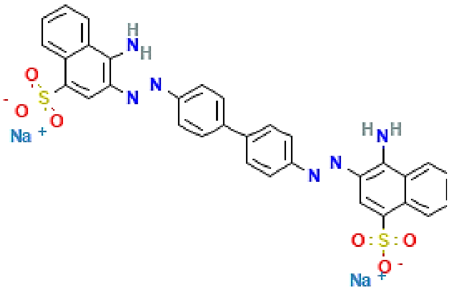	$C_{32}H_{22}N_6Na_2O_6S_2$	696.7	C.I. Direct Red 28	502	573-58-0
Blue RL	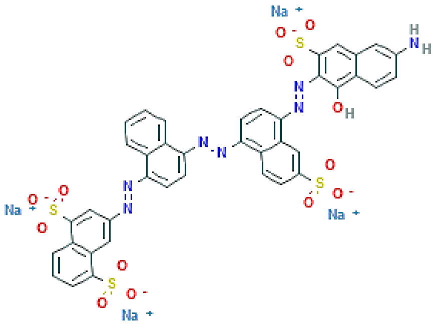	$C_{40}H_{23}N_7Na_4O_{13}S_4$	1029.9	C. I. Direct Blue 71	588	4399-55-7
Scarlet 4BS	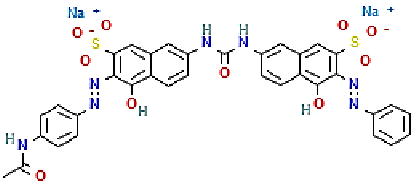	$C_{35}H_{25}N_7Na_2O_{10}S_2$	813.7	direct red 23<br>C.I. 29160	507	3441-14-3
Red 8B	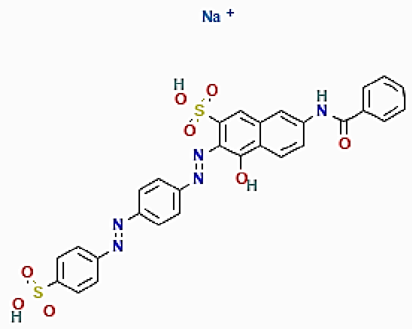	$C_{29}H_{21}N_5NaO_8S_2$+	654.6	C.I. Direct Red 81	510	2610-11-9

Violet R	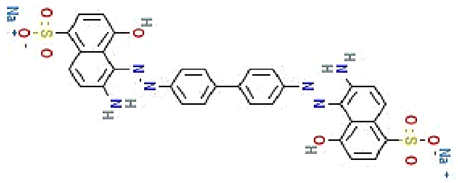	$C_{32}H_{22}N_6Na_2O_8S_2$	728.7	C.I. Direct Violet 1 (VAN)	532	2586-60-9
Brown RC	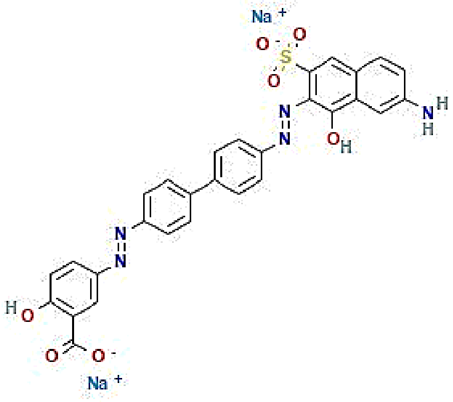	$C_{29}H_{19}N_5Na_2O_7S$	627.5	Direct fast brown C.I. 22311	460	2429-82-5
Turquoise GLL	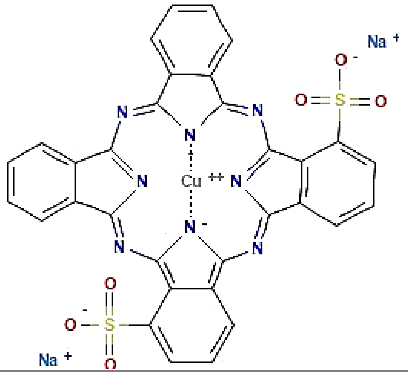	$C_{32}H_{14}CuN_8Na_2O_6S_2$	780.2	Lionol blue GS C.I. 74180	600	1330-38-7
Blue 4GL	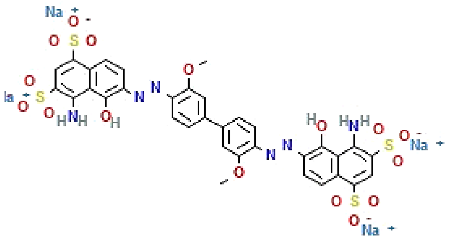	$C_{34}H_{28}N_6NaO_{16}S_4$+	927.9	Chicago Sky Blue 6B, Direct Blue 1 C.I. 24410	605	2610-05-1

Black Meta	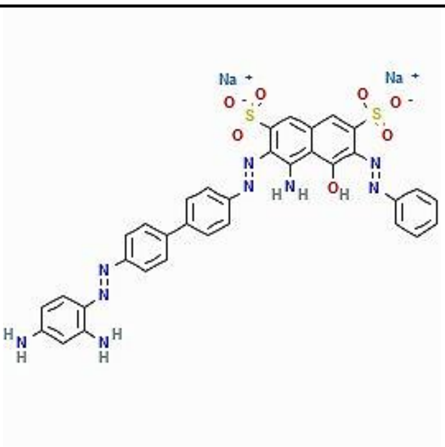	$C_{34}H_{25}N_9Na_2O_7S_2$	781.7	C.I. Direct Black 38	520	1937-37-7

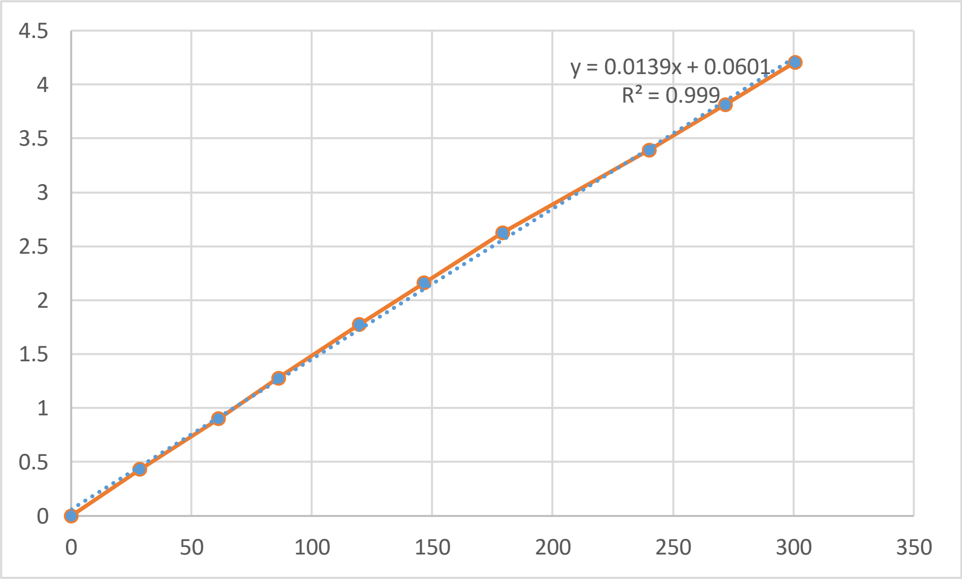

Figure 1. Calibration curve of Orange GRLL dye (30-300) mg/L, pH 7, 25 °C.

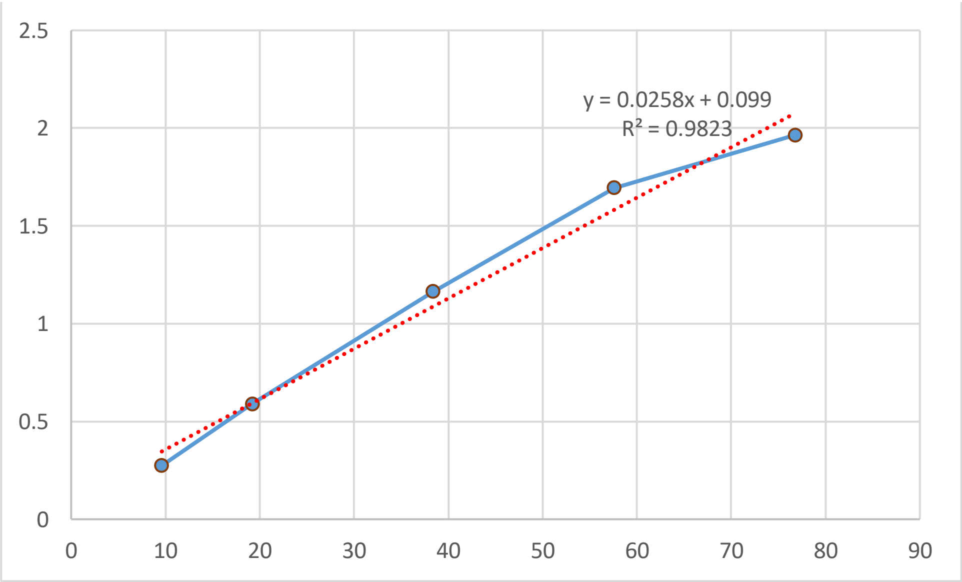

Figure S2 Calibration curve of Blue 3B dye (10-80) mg/L, pH 7, 25 °C.

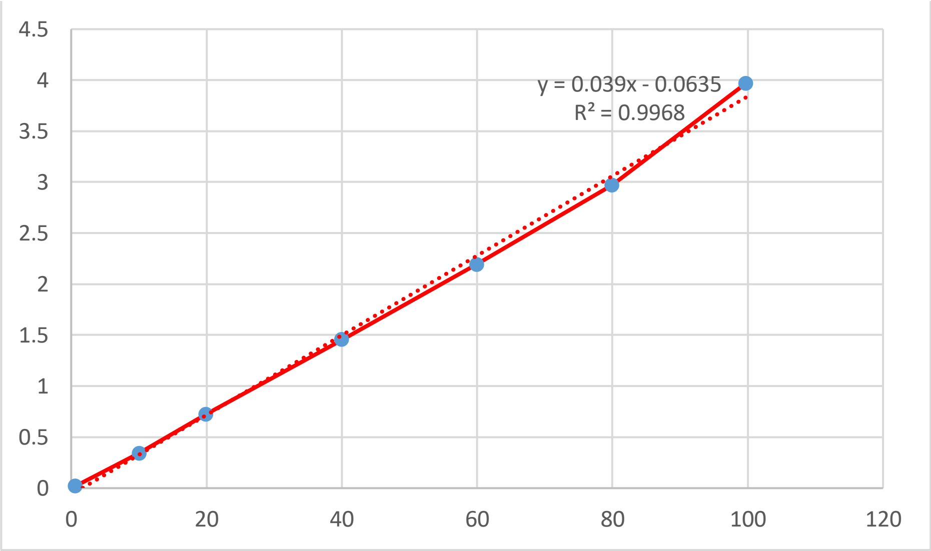

Figure S3 Calibration curve of Congo Red dye (10-100) mg/L, pH 7, 25 ^{o}C.

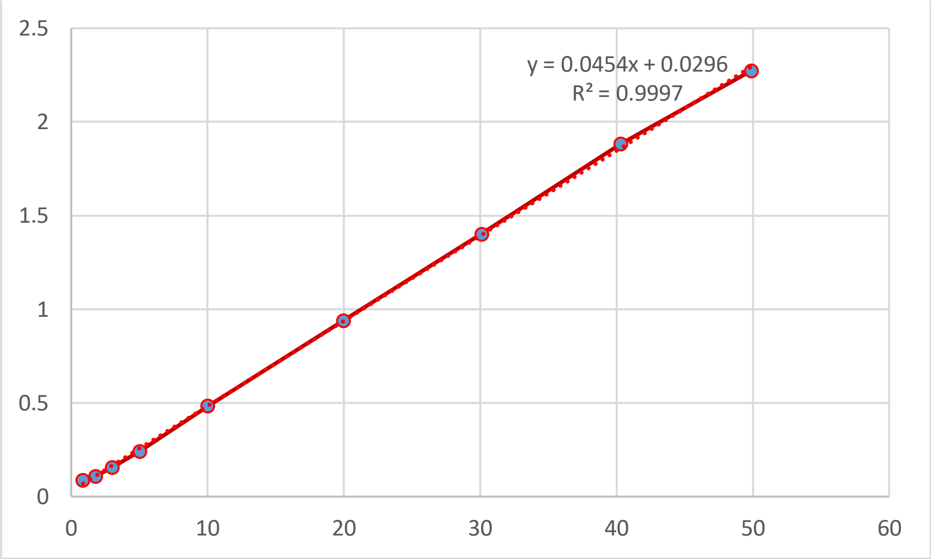

Figure S4 Calibration curve of Red 8B dye (1-50) mg/L, pH 7, 25 ^{o}C.

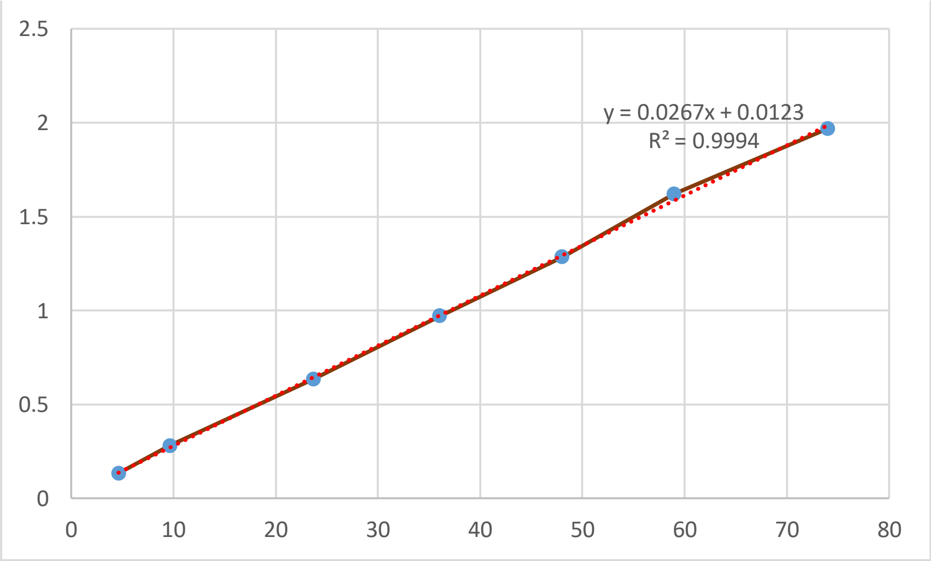

Figure S5 Calibration curve of Scarlet 4BS (5-75) mg/L, pH 7, 25 ^{o}C.

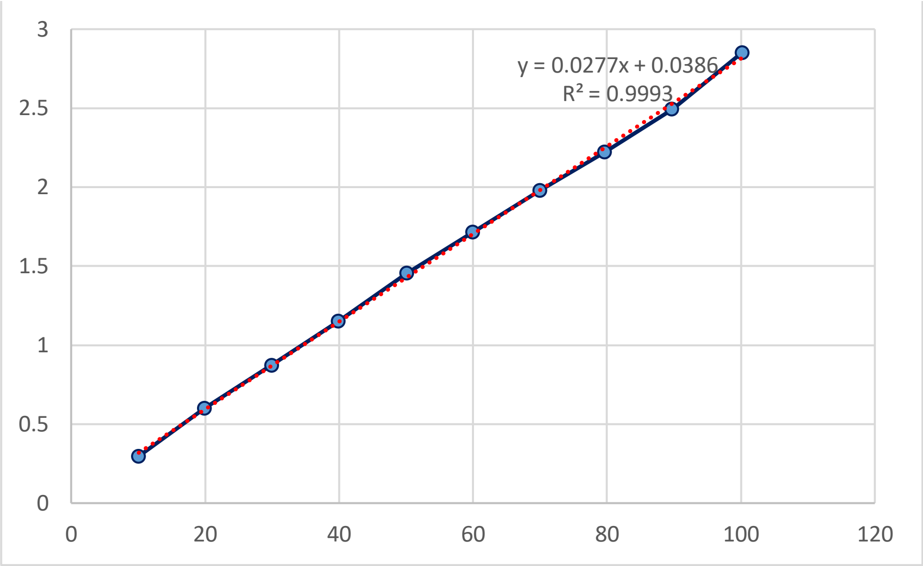

Figure S6 Calibration curve of Violet R (10-100) mg/L, pH 7, 25 °C.

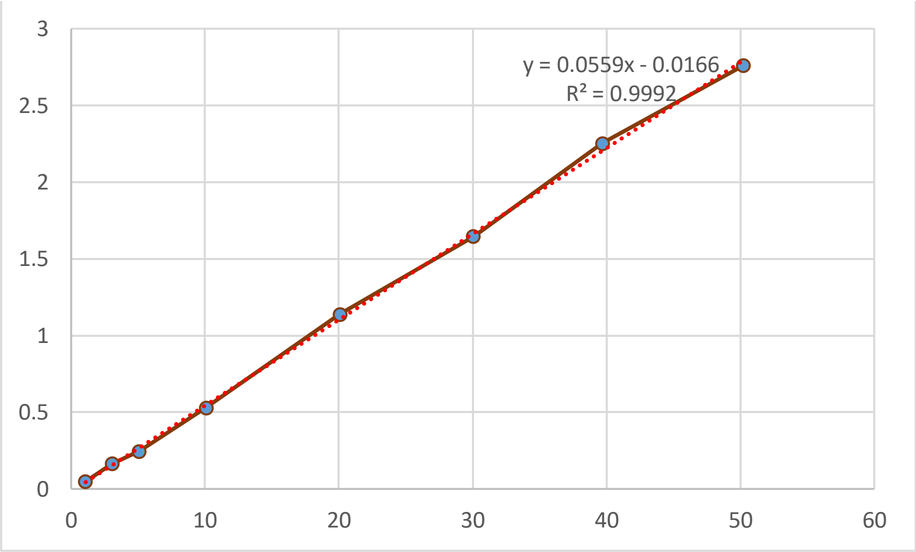

Figure S7 Calibration curve of Brown RC (1-50) mg/L, pH 7, 25 °C.

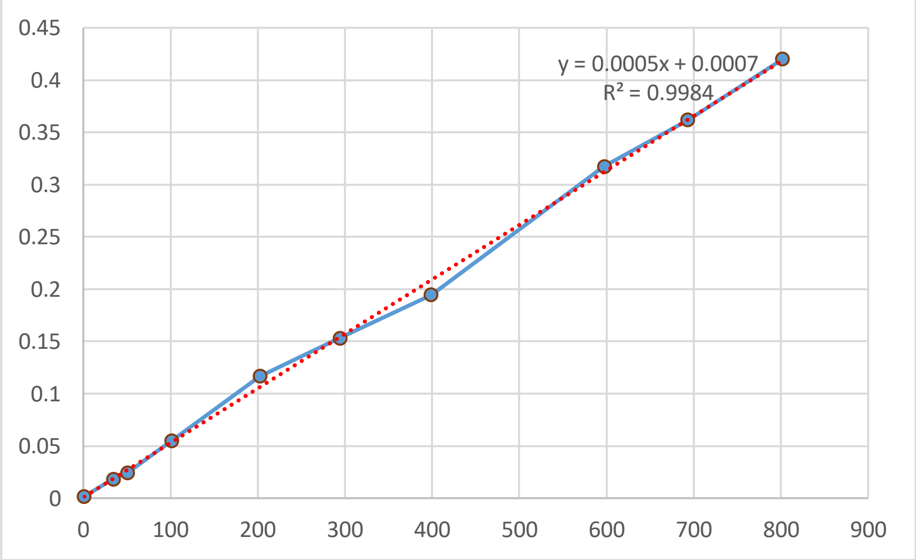

Figure S8 Calibration curve of Turquoise Blue GLL (10-800) mg/L, pH 7, 25 °C.

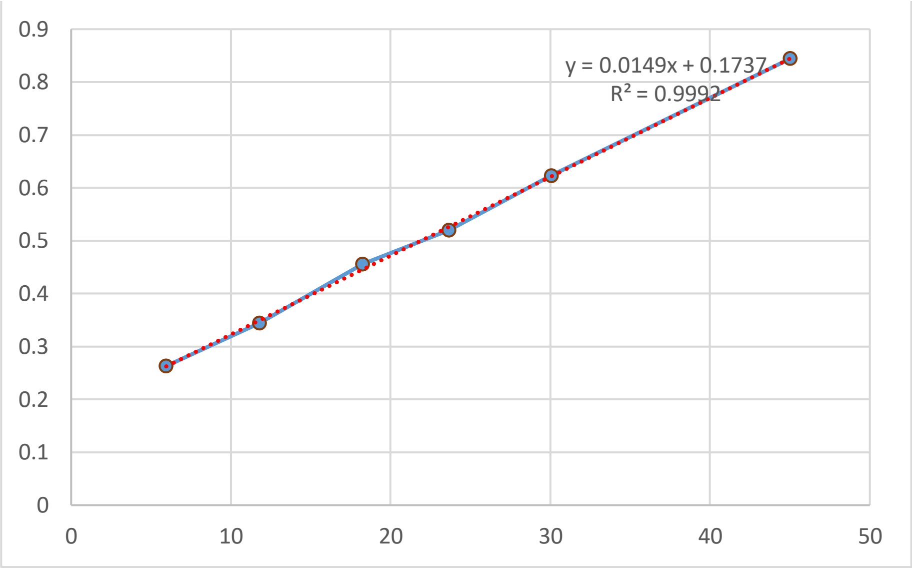

Figure S9 Calibration curve of Blue RL (5-45) mg/L, pH 7, 25 ºC.

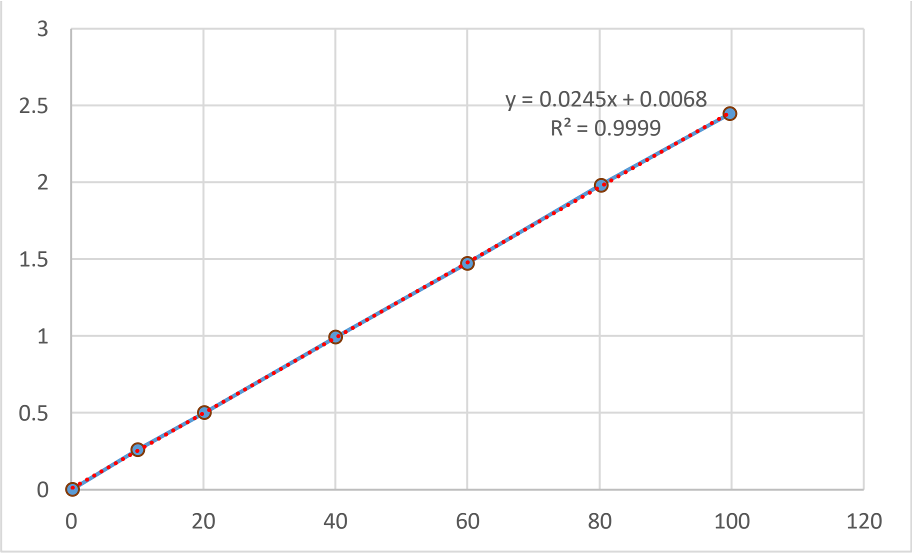

Figure S10 Calibration curve of Yellow G (1-100) mg/L, pH 7, 25 ºC.

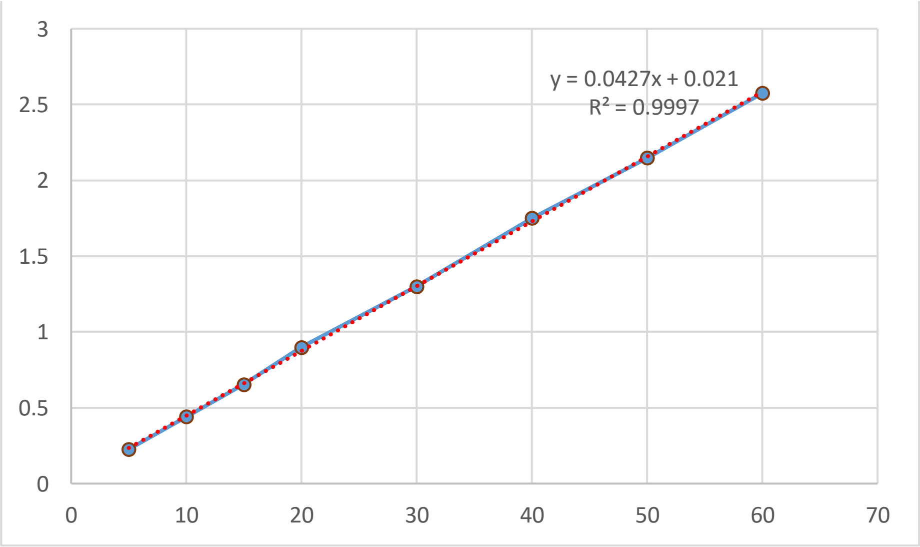

Figure S11 Calibration curve of Blue 4GL (5-60) mg/L, pH 7, 25 ºC.

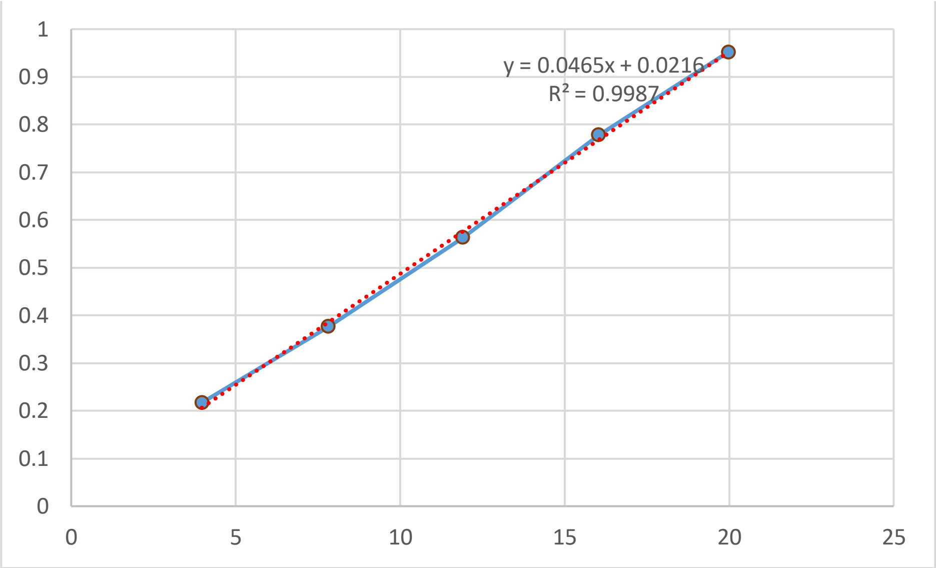

Figure S12 Calibration curve of Black meta (5-20) mg/L, pH 7, 25 °C.

Table S2 Freundlich ISOTHERM for SBR DATA

	log Ce (mg/L)	log(X/m) (mg/g)
BLUE RL	0.859	0.863
	1.095	1.049
	1.224	1.151
	1.271	1.187
	0.971	0.951
BLUE 4 GL	0.497	1.277
	0.716	1.340
	0.888	1.408
	1.007	1.445
	1.128	1.487
SCARLET 4BS	0.617	0.958
	0.704	1.042
	0.888	1.188
	0.969	1.304
	1.168	1.524
BROWN RC	0.813	0.921
	0.920	1.005
	1.150	1.147
	1.237	1.236
	1.280	1.277
Congo red	0.980	0.623
	1.064	0.743
	1.084	0.890
	1.101	1.010
	1.130	1.141
VIOLET R	1.337	0.984
	1.378	1.047
	1.398	1.099
	1.438	1.183
	1.493	1.272
YELLOW G	1.775	1.293

	1.801	1.429
	1.836	1.581
	1.848	1.707
	1.859	1.838
TURQUOISE BLUE GLL	0.727	1.681
	1.036	1.759
	1.355	1.817
	1.609	1.885
	1.773	1.932
YELLOW RL	0.757	2.115
	1.079	2.186
	1.327	2.241
	1.704	2.324
	1.586	2.298
RED 8B	0.869	2.099
	1.183	2.209
	1.016	2.147
	1.615	2.319
	1.883	2.398
BLUE 3B	1.344	2.236
	1.491	2.335
	1.595	2.429
	1.716	2.476
	1.817	2.529
BLACK META	1.465	2.162
	1.483	2.257
	1.500	2.382
	1.540	2.560
	1.593	2.848
ORANGE GLL	2.165	2.068
	2.179	2.141
	2.220	2.335
	2.246	2.503
	2.194	2.215

Table S3 Freundlich ISOTHERM for WBR DATA

	log Ce (mg/L)	log(X/m) (mg/g)
Congo red	0.579	0.508
	0.649	0.575
	0.788	0.709
BLUE RL	1.339	0.873
	1.507	0.980
	1.577	1.024
	1.652	1.072
VIOLET R	1.759	0.633
	1.759	0.689
	1.773	0.728
	1.806	0.935
	1.796	0.857

	1.782	0.773
SCARLET 4BS	1.474	0.864
	1.521	0.948
	1.591	1.093
	1.689	1.255
	1.628	1.155
BROWN RC	1.162	0.753
	1.222	0.831
	1.358	1.004
	1.437	1.015
	1.465	1.144
	1.488	1.256
BLUE 4GL	0.821	1.287
	0.854	1.320
	0.900	1.370
	0.961	1.409
	1.054	1.533
YELLOW G	1.894	1.120
	1.922	1.271
	1.946	1.479
	1.978	1.703
	2.011	1.910
BLACK META	2.090	1.203
	2.109	1.332
	2.123	1.500
	2.123	1.624
	2.142	1.798
BLUE 3B	1.437	2.344
	1.540	2.467
	1.656	2.635
	1.754	2.775
	1.834	2.892
RED 8B	1.390	2.154
	1.507	2.265
	1.642	2.371
	1.689	2.444
	1.764	2.522
TURQUOISE BLUE GLL	2.076	1.618
	2.230	1.730
	2.309	1.819
	2.356	1.870
	2.557	2.076
ORANGE GLL	2.529	1.819
	2.533	1.897
	2.580	1.992
	2.683	2.143
	2.771	2.277
YELLOW RL	2.631	2.844
	2.659	2.848
	2.720	2.856

	2.771	2.862
	2.809	2.867

Table S4 Langmiur ISOTHERM for SBR DATA

	m/X (g/mg)	1/Ce (L/mg)
ORANGE GLL	0.005	0.002
	0.006	0.005
	0.006	0.006
	0.007	0.008
YELLOW G	0.014	0.014
	0.015	0.023
	0.016	0.032
	0.017	0.052
BLACK META	0.027	0.005
	0.036	0.007
	0.033	0.006
	0.029	0.006
BLUE 3B	0.016	0.003
	0.026	0.005
	0.045	0.006
	0.034	0.005
VIOLET R	0.016	0.005
	0.140	0.008
	0.068	0.006
	0.112	0.006
YELLOW RL	0.018	0.258
	0.048	0.258
	0.178	0.258
	0.158	0.258
TURQUOISE BLUE GLL	0.017	0.011
	0.024	0.014
	0.045	0.016
	0.189	0.021
	0.166	0.019
RED 8B	0.033	0.052
	0.037	0.066
	0.040	0.079
	0.046	0.106
BLUE 4 GL	0.074	0.030
	0.129	0.039
	0.194	0.045
	0.272	0.051
	0.322	0.054
SCARLET 4BS	0.069	0.029
	0.108	0.050
	0.127	0.069
	0.206	0.092
	0.247	0.110
BROWN RC	0.053	0.052

	0.123	0.097
	0.155	0.119
	0.071	0.073
Congo red	0.079	0.069
	0.083	0.128
	0.088	0.176
	0.104	0.240
BLUE RL	0.054	0.621
	0.060	0.621
	0.078	0.621
	0.136	0.621

Table S5 Langmiur ISOTHERM for WBR DATA

	1/Ce (L/mg)	m/X (g/mg)
BLUE 3B	0.023	0.000
	0.033	0.001
	0.041	0.003
	0.053	0.002
	0.057	0.003
TURQUOISE BLUE GLL	0.001	0.001
	0.003	0.004
	0.005	0.005
	0.007	0.006
BLACK META	0.012	0.010
	0.013	0.020
	0.013	0.029
	0.013	0.039
YELLOW G	0.016	0.007
	0.017	0.012
	0.017	0.019
	0.018	0.032
	0.020	0.042
RED 8B	0.030	0.002
	0.037	0.002
	0.044	0.003
	0.052	0.004
	0.061	0.005
SCARLET 4BS	0.031	0.036
	0.038	0.052
	0.040	0.063
	0.043	0.072
	0.048	0.086
YELLOW RL	0.003	0.001
	0.004	0.004
	0.048	0.006
ORANGE GLL	0.002	0.003
	0.005	0.007
	0.006	0.010
BROWN RC	0.046	0.035

	0.051	0.050
	0.053	0.062
	0.070	0.077
	0.078	0.094
BLUE 4 GL	0.114	0.019
	0.125	0.021
	0.138	0.024
	0.176	0.030
	0.179	0.032
VIOLET R	0.025	0.074
	0.025	0.098
	0.026	0.119
	0.026	0.131
	0.027	0.150
BLUE RL	0.032	0.208
	0.036	0.208
	0.040	0.208
	0.048	0.208
	0.061	0.208
Congo red	0.196	0.125
	0.246	0.157
	0.267	0.171
	0.299	0.191
